# Extreme seascape drives local recruitment and genetic divergence in brooding and spawning corals in remote north‐west Australia

**DOI:** 10.1111/eva.13033

**Published:** 2020-06-22

**Authors:** Jim N. Underwood, Zoe Richards, Oliver Berry, Daniel Oades, Azton Howard, James P. Gilmour

**Affiliations:** ^1^ Australian Institute of Marine Science Indian Oceans Marine Research Centre, Crawley Perth WA Australia; ^2^ Western Australian Marine Science Institution Indian Ocean Marine Research Centre Crawley WA Australia; ^3^ Trace and Environmental DNA Laboratory School of Molecular and Life Sciences Curtin University Bentley WA Australia; ^4^ Department of Aquatic Zoology Western Australian Museum Welshpool WA Australia; ^5^ CSIRO Oceans and Atmosphere Indian Oceans Marine Research Centre, Crawley Perth WA Australia; ^6^ Bardi Jawi Rangers Kimberley Land Council Broome WA Australia

**Keywords:** *Acropora aspera*, conservation genomics, *Isopora brueggemanni*, marine reserve networks, population connectivity, single nucleotide polymorphism

## Abstract

Management strategies designed to conserve coral reefs threatened by climate change need to incorporate knowledge of the spatial distribution of inter‐ and intra‐specific genetic diversity. We characterized patterns of genetic diversity and connectivity using single nucleotide polymorphisms (SNPs) in two reef‐building corals to explore the eco‐evolutionary processes that sustain populations in north‐west Australia. Our sampling focused on the unique reefs of the Kimberley; we collected the broadcast spawning coral *Acropora aspera* (*n* = 534) and the brooding coral *Isopora brueggemanni* (*n* = 612) across inter‐archipelago (tens to hundreds of kilometres), inter‐reef (kilometres to tens of kilometres) and within‐reef (tens of metres to a few kilometres) scales. Initial analysis of *A. aspera* identified four highly divergent lineages that were co‐occurring but morphologically similar. Subsequent population analyses focused on the most abundant and widespread lineage, *Acropora* asp‐c. Although the overall level of geographic subdivision was greater in the brooder than in the spawner, fundamental similarities in patterns of genetic structure were evident. Most notably, limits to gene flow were observed at scales <35 kilometres. Further, we observed four discrete clusters and a semi‐permeable barrier to dispersal that were geographically consistent between species. Finally, sites experiencing bigger tides were more connected to the metapopulation and had greater gene diversity than those experiencing smaller tides. Our data indicate that the inshore reefs of the Kimberley are genetically isolated from neighbouring oceanic bioregions, but occasional dispersal between inshore archipelagos is important for the redistribution of evolutionarily important genetic diversity. Additionally, these results suggest that networks of marine reserves that effectively protect reefs from local pressures should be spaced within a few tens of kilometres to conserve the existing patterns of demographic and genetic connectivity.

## 1 INTRODUCTION

Species fitness depends on the standing stock of genetic variation (Fisher, 1930; Reed & Frankham, 2003); populations with high genetic diversity are often more resilient than less diverse populations (Hughes, Inouye, Johnson, Underwood, & Vellend, 2008; Palumbi, Barshis, Traylor‐Knowles, & Bay, 2014). Therefore, managers of biological resources threatened by climate change need to consider not only the distribution of genetic diversity within (Carvalho et al., 2017) and between (Duffy, Godwin, & Cardinale, 2017) species, but also the processes that create and maintain that diversity (Calosi, De Wit, Thor, & Dupont, 2016). However, the integration of genetic metrics into conservation planning in marine systems, especially across multiple species, is still in its infancy (Cook & Sgrò, 2017; Magris, Treml, Pressey, & Weeks, 2016; Nielsen, Beger, Henriques, Selkoe, & von der Heyden, 2017). Given that coral reefs are declining rapidly due to extreme ocean temperatures, acidification and local anthropogenic disturbances (Hoegh‐Guldberg et al., 2007; Hoey et al., 2016; Hughes et al., 2018), their effective management requires knowledge of both the ecological drivers of population replenishment (Magris, Pressey, Weeks, & Ban, 2014) and their evolutionary resilience to changing climatic conditions (Drury, 2020; Matz, Treml, Aglyamova, & Bay, 2018; Quigley, Bay, & van Oppen, 2019; van Woesik, 2017). Specifically, this eco‐evolutionary understanding should be incorporated into decisions about prioritization, size and spacing of networks of marine reserves (Lamb, Williamson, Russ, & Willis, 2015; McCook et al., 2010; Mellin, Aaron MacNeil, Cheal, Emslie, & Julian Caley, 2016).

The coral reef systems of the Kimberley in north‐west Australia are a biophysically unique centre of coral biodiversity at the southern margin of the East Indies Coral Triangle (Wilson, 2013) and are among the world's most remote and least degraded ecosystems (Halpern et al., 2008). This region may also play an important role as a tropical refuge for photosymbiotic benthic fauna (Richards et al., 2019). However, some inshore Kimberley reefs bleached for the first time in 2016 (Gilmour et al., 2019; Hughes et al., 2018), highlighting that even these reefs that are far from urban centres and agricultural influences are susceptible to global warming. Macrotides (up to 12 m) combine with complex geomorphology to create powerful currents (>1m s^−1^; Ivey et al., 2016), which could be either strong conduits or barriers to dispersal of larvae among local populations. These reefs also experience large variations in temperature, turbidity, nutrient concentrations and aerial exposure (Jones, Patten, et al., 2014; Richards, Garcia, Wallace, Rosser, & Muir, 2015; Schoepf, Stat, Falter, & McCulloch, 2015; Wilson, 2013). Limited cross‐shelf and long‐shore circulation (D'Adamo, Fandry, & Domingues, 2009; Treml & Halpin, 2012) suggest inshore populations are isolated from others in the region. Theory predicts that physical isolation coupled with strong selection pressures from extreme environmental heterogeneity will produce unique patterns of inter‐ and intra‐specific genetic diversity and structure in populations (Felsenstein, 1976). This prediction has not been well tested in the Kimberley for reef‐building corals, but records of new species (D. Jones, Patten, Bryce, Fromont, & Moore, 2014; Richards et al., 2015) and unique species/habitat associations (Richards, Bryce, Bryce, & Bryce, 2013) are beginning to substantiate this expectation.

Knowledge of larval connectivity is fundamental to spatial planning for coral reef conservation because it is a key ecological driver of population replenishment and recovery after disturbance (Cowen & Sponaugle, 2009). There is currently limited knowledge of metapopulation dynamics of most reefs and species, and even less understanding of how to integrate connectivity information into ecosystem management (Magris et al., 2014; Underwood, Wilson, Ludgerus, & Evans, 2013). Because genetic divergence among individuals and populations accumulates over multiple generations through genetic drift and differential selection when inter‐breeding is restricted, spatial analysis of genetic structure is a pivotal method for measuring connectivity (Hedgecock, Barber, & Edmands, 2007).

It is often difficult to resolve species boundaries in corals due to their morphological plasticity and propensity for hybridization (Ladner & Palumbi, 2012; Richards & Hobbs, 2015; Schmidt‐Roach, Miller, Lundgren, & Andreakis, 2014; Willis, 1990). A growing body of evidence suggests that cryptic diversity exists within previously well‐known species of corals, and cryptic lineages in north‐west Australia have been shown to be associated with habitat (Thomas et al., 2020; Underwood, Richards, Miller, Puotinen, & Gilmour, 2018), timing of reproduction (Gilmour, Underwood, Howells, Gates, & Heyward, 2016; Rosser, 2015,2016; Rosser, Edyvane, Malina, Underwood, & Johnson, 2020; Rosser et al., 2017) or unknown mechanisms (Richards, Berry, & Oppen, 2016; Thomas et al., 2014). These studies highlight that a rigorous assessment of cryptic diversity needs to become the critical first step in population genetic analyses of corals (Sheets, Warner, & Palumbi, 2018).

This study characterized the genetic diversity and connectivity within and among populations of *Acropora aspera* (Dana, 1846) and *Isopora brueggemanni* (Brook, 1893) from Kimberley reefs of north‐west Australia. Both these species are widespread branching corals that provide the three‐dimensional habitat for many coral reef organisms throughout the Indo‐Pacific. Although they both belong to the family Acroporidae, these two species differ in modes of reproduction. *Acropora aspera* is a broadcast spawner, releasing eggs and sperm into the water column where fertilization and larval development occur. The larvae spend a few days in the plankton before they are competent to settle (Appendix A). In contrast, *I. brueggemanni* is a brooder. Fertilization and larval development occur within the polyp before larvae are released at an advanced developmental stage capable of settling within a few hours (Appendix A). Both species are listed as vulnerable on the IUCN Red List of Threatened Species based on their geographic range and their susceptibility to bleaching and disease (Aeby et al., 2014; Richards et al., 2008).

Here, we investigated the eco‐evolutionary processes that sustain the metapopulations of *A. aspera* and *I. brueggemanni* in north‐west Australia by genotyping thousands of single nucleotide polymorphisms (SNPs) isolated from across their genomes. We first tested for cryptic diversity within samples identified as *A. aspera* or *I. brueggemanni*. We then measured the spatial distribution of genetic diversity at inter‐archipelago (tens to hundreds of kilometres), inter‐reef (kilometres to tens of kilometres) and within‐reef (hundreds of metres to kilometres) scales to determine the relative strength of genetic connections. Finally, we explored key seascape drivers of metapopulation structure by testing whether heterogeneity in environmental factors such as temperature, turbidity and tide was associated with genetic differentiation and diversity of local coral populations.

## 2 MATERIALS AND METHODS

### 2.1 Sampling design

**FIGURE 1:**
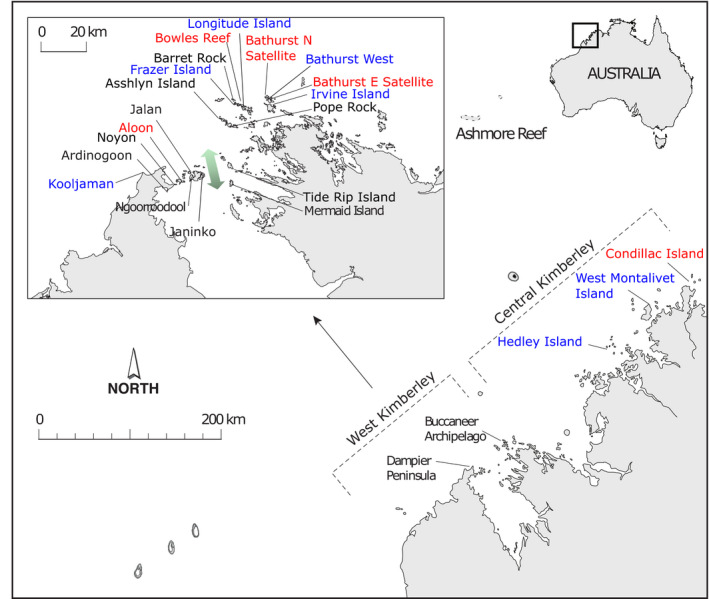
Map of *Acropora aspera* and *Isopora brueggemanni* collections from the west and central inshore Kimberley and Ashmore Reef in north‐west Australia. Insert shows locations of detailed collections from the Dampier Peninsula and the Buccaneer Archipelago. Black text indicates sites where both species were collected, red text indicates sites where only *A. aspera* was collected, and blue text indicates sites where only *I. brueggemanni* was collected. Double‐headed arrow indicates the tidally driven current through the Sunday Strait which separates the Dampier Peninsula from the Buccaneer Archipelago

We sampled a range of spatial scales (Figure 1). At the broadest scale, we collected corals separated by tens to hundreds of kilometres from different bioregions (offshore Ashmore Reef versus inshore Kimberley) and archipelagos; archipelagos are hereafter referred to as the geographically separate systems of Ashmore Reef, Dampier Peninsula, Buccaneer Archipelago and central Kimberley. At the intermediate scale, we sampled multiple reefs separated by kilometres to tens of kilometres through detailed collections from the Buccaneer Archipelago and the Dampier Peninsula in the west of the Kimberley. At the fine scale, we recorded the location of colonies with GPS and for the brooding coral sampled a replicate site (separated from the first site by ~500 m) within three reefs of the Buccaneer Archipelago.

We collected samples by walking on exposed platforms at spring low tides and removing one‐centimetre fragments from coral colonies. Fragments were preserved in 100% ethanol. We photographed colonies and collected representative voucher specimens for taxonomic verification. We collected 534 *Acropora aspera* samples from 15 sites (between 24 and 83 colonies per site; Table 1) and 612 *Isopora brueggemanni* samples from 18 sites (between 20 and 60 samples per site; Table 2).

**TABLE 1:** Numbers of samples and unique colonies (genets) of *Acropora aspera* collected from sites from the Kimberley coast and Ashmore Reef in north‐west Australia. *N* (all) is the total number in the entire collection of *Acropora aspera*. *N* (asp‐c) is the total number of samples identified as *Acropora* asp‐c, *N*g (asp‐c) is the number of genets of *Acropora* asp‐c, and *N*g:*N* (asp‐c) is the genotypic richness of *Acropora* asp‐c

Region	Site	*N* (all)	*N (*asp‐c)	*Ng (*asp‐c)	*N*g:*N* (asp‐c)
Ashmore	Ashmore_Reef	34	7	5	0.71
Central Kimberley	Condilac_Is	32	10	10	1.00
Buccaneer Archipelago	Bathurst_N_Sat	27	12	3	0.25
	Bathurst_E_Sat	30	30	12	0.40
	Bowles_Rock	30	30	10	0.33
	Barret_Rock	31	30	19	0.63
	Asshlyn_Is	61	58	36	0.62
	Pope_Is	30	30	10	0.33
	Tide_Rip	31	27	20	0.74
	Mermaid_Is	30	29	15	0.52
Dampier Peninsula	Janinko	31	28	18	0.64
	Ngoorroodool	32	2	2	1.00
	Aloon	24	9	9	1.00
	Noyon	28	0	—	—
	Ardinoogoon	83	0	—	—
	TOTAL	534	302	169	0.63

**TABLE 2:** Numbers of samples and unique colonies (genets) of *Isopora brueggemanni* collected from sites from the Kimberley coast and Ashmore Reef in north‐west Australia. N is the total numbers of samples, Ng is the number of genets, and Ng:N is the genotypic richness

Region	SITE	*N*	*Ng*	*N*g:*N*
Ashmore	Ashmore_Reef	29	29	1.00
Central Kimberley	West_Montalivet	32	25	0.78
	Hedley_Is	28	11	0.39
Buccaneer Archipelago	Irvine_Is	27	27	1.00
	Bathhurst_W_1	28	28	1.00
	Bathhurst_W_2	20	20	1.00
	Longitude_Is	29	29	1.00
	Frazer_Is	31	30	0.97
	Barret_Rock	29	27	0.93
	Asshlyn_Is	31	30	0.97
	Pope_Is_1	31	30	0.97
	Pope_Is_2	31	30	0.97
	Tide_Rip_Is	31	29	0.94
	Mermaid_Is_1	30	30	1.00
	Mermaid_Is_2	30	26	0.87
Dampier Peninsula	Janinko	29	26	0.90
	Ngoorroodool	20	20	1.00
	Jalan	30	30	1.00
	Noyon	30	28	0.93
	Ardinoogoon	30	25	0.83
	Kooljaman	31	31	1.00
	TOTAL	606	561	0.93

### 2.2 SNP development, QC and diversity

We extracted genomic DNA from coral specimens using a salting‐out protocol modified from Cawthorn, Steinman, and Witthuhn (2011) and purified with Zymo‐Spin I‐96 Filter plates. Genome‐wide SNP data were generated using the next‐generation sequencing platform and the DArT‐seq protocol. DArT‐seq is similar to other site‐associated restriction enzyme‐based library preparation methods (e.g. RAD‐seq) and is a widely applied approach for exploring population genetic structure in species that lack genome assemblies (DiBattista et al., 2017; Pazmino, Maes, Simpfendorfer, Salinas‐de‐Leon, & van Herwerden, 2017; Thomas et al., 2020). Sequencing was carried out on an Illumina HiSeq 2,500 using 75‐cycle single‐end reads. Raw reads were processed using DArT's proprietary variant calling pipeline, DArTsoft‐14. The call quality of the initial SNP data set was further assured by setting a cut‐off of read depth per locus (coverage) <7, call rate >0.35 and minimum allele frequency >0.00075 for *Isopora* and >0.0017 for *Acropora* (further details of DArT‐seq protocol in Appendix B). This development phase indicated the presence of highly divergent genetic lineages within *A. aspera*. We subsequently applied a stringent filter to the data to isolate loci suitable for inter‐specific analysis. From the primary data set of 34,304 SNPs, we used adegenet (Jombart, 2008) and the dartR package (Gruber, Georges, Unmack, & Berry, 2017) to filter using call rate >0.95, coverage >20, minimum allele frequency >0.05 and max heterozygosity <0.75. In addition, we used the reproducibility statistic to filter out all loci with <0.999 correct calls across individuals. These filters were chosen to minimize genotyping noise such as null alleles brought about by differences in the target sequences among divergent genetic groups. The final filtered *A. aspera* data set comprised of 585 SNPs. However, to make sure this stringent set of loci did not bias differentiation estimates, we also conducted our inter‐specific analysis with relaxed filters (call rate >0.80, coverage >20, a minimum allele frequency >0.01 and reproducibility >0.999). We did not filter for Hardy–Weinberg or gametic‐phase disequilibrium at this stage of the analysis because large (potentially inter‐specific) divergence would be associated with such disequilibrium, and removal of such markers would likely limit power of the analyses. Seven individuals with more than 15% missing data were removed.

We identified four distinct lineages in the *A. aspera* samples that often occurred in sympatry (see results). Due to low sample sizes in three of the four lineages, we only conducted population‐level analyses on the most common and widespread lineage (*Acropora* asp‐c). To this end, we recalculated the descriptive statistics across all SNP loci for those samples identified as *Acropora* asp‐c with the same filters and methods as for the entire *A. aspera* collection except we relaxed the reproducibility (>0.98) and call rate (>0.90) thresholds. This filtering resulted in 3,472 loci. We then filtered out loci that significantly departed from Hardy–Weinberg equilibrium and gametic‐phase equilibrium with R packages dartR, SNPassoc (Gonzalez et al., 2007), adegenet and pegas (Gonzalez et al., 2007; Paradis, 2010). We tested for disequilibrium separately for each sampling site with more than 15 samples (*N* = 5 sites). For Hardy–Weinberg testing, we removed 343 loci that showed departures from expectations at *p* < .05 in three or more (out of five) sites. For gametic‐phase disequilibrium, we removed 294 loci with *r* values >0.8 at three or more sites. In the remaining 2,898 SNPs, we identified loci possibly affected by selection with OutFLANK v0.1 (Whitlock & Lotterhos, 2015) using 5% left and right trim for the null distribution of *F*_ST_, minimum heterozygosity for loci of 0.1 and a 5% false discovery rate (*q* value). Four loci were identified as outliers, and these were removed from subsequent analyses resulting in final data set of 2,894 loci.

There was no indication of cryptic diversity in *I. brueggemanni*, and we filtered the primary data set (*n* = 23, 165 loci) using the same criteria as for *Acropora* asp‐c. This resulted in 2,946 loci. We then filtered out loci that exhibited significant Hardy–Weinberg and linkage disequilibrium at each sampling site (*n* = 21). For Hardy–Weinberg disequilibrium, we removed 133 loci that showed departures from expectations at *p* < .05 in five or more of the 21 sites. For linkage disequilibrium, we removed 681 loci with *r* values >0.8 among five or more sites. These filters resulted in 2,132 SNPs. Six *I. brueggemanni* individuals with more than 15% missing data were removed. We identified putative loci affected by selection as for the *Acropora* asp‐c analysis. Initial analysis using the entire data set did not detect any outliers, but when OutFLANK was applied to the inshore data only, seven loci were identified as outliers and were removed from subsequent analyses resulting in final data set of 2,125 loci.

After removal of clones (Appendix C), we calculated summary statistics of the final data sets in GenAlEx v6.5 (Peakall & Smouse, 2006), including number of positive calls (*N*), genotypic richness (the ratio of number of genets to total number of samples), observed heterozygosity (*H*_O_), gene diversity measured as unbiased expected heterozygosity (*H*_E_) and fixation index (*F*_IS_) at each site and averaged across sites (±standard error).

### 2.3 Cryptic diversity

We tested for the presence of cryptic diversity within our collections with a cluster analysis that identified the optimal number of genetic clusters (*K*) and membership coefficients (*q*) of each colony to a range of clusters with the Bayesian software STRUCTURE v2.3 (Pritchard, Stephens, & Donnelly, 2000). Mean and variance of log‐likelihoods and posterior probabilities of the number of clusters from K = 1 to 8 were inferred using correlated allele frequency with admixture model and burn‐in of 10,000 and then 100,000 MCMC repetitions. We checked convergence of algorithms by assessing the stability of runtime **α** and Ln likelihood after burn‐in, the variability in individual assignment proportions and the similarity score calculated with the online program CLUMPAK (Kopelman, Mayzel, Jakobsson, Rosenberg, & Mayrose, 2015) from ten replicate runs. As recommended by Wang (2016), we used a separate **α** for each population and applied an initial value of **α** = 0.25 (1/*K* ascertained from exploratory runs), and all other parameters were set as default values. CLUMPAK was used to summarize and graphically present the STRUCTURE results as well as to calculate optimal *K* using the Δ*K* method of Evanno, Regnaut, and Goudet (2005). We also considered alternative *K* values in addition to Δ*K* including Ln(Pr(X|*K*) values to identify the k for which Pr(*K* = *k*) is highest (Pritchard & Wen, 2004) and chose the *K* that best described the data and addressed a priori questions and expectations (see Meirmans, 2015; Pritchard & Wen, 2004). When divergent samples were detected (e.g. cryptic diversity or strong geographic divergence), we performed subsequent runs excluding these divergent samples to increase clustering accuracy among the genetically coherent samples (see Janes et al., 2017).

The initial analysis in STRUCTURE identified four divergent and sympatric lineages of *A. aspera* (see Results). We gauged the relative divergence among versus within these lineages by estimating the genetic relationships among individuals with principal coordinate analysis (PCoA) in GenAlEx v6.5. PCoA takes a simple multi‐ordination approach calculated from a codominant genotypic distance among pairs of samples and does not incorporate any a priori information or assumptions of equilibrium. Therefore, PCoA provides a complimentary analysis to estimate the number and membership of clusters to the sophisticated Bayesian approach of STRUCTURE. We used the standardized distance option for the PCoA. We also calculated pairwise FST between lineages and the number of private alleles (*P*_A_) in each lineage in GenAlEx to further estimate the magnitude of differentiation among lineages.

### 2.4 Inter‐archipelago to inter‐reef population structure

We examined the population genetic structure at broad scales with STRUCTURE, PCoA and AMOVA using samples from the entire collections of the *Acropora* asp‐c lineage and *I. brueggemanni*. STRUCTURE and PCoA were run with the same parameters as in the tests for cryptic diversity. However, we ran STRUCTURE from *K = *1 to 10 for *I. brueggemanni* because geographic clusters continued to segregate at *K* >8. We assessed the genetic relationships among corals that were obscured by divergent samples by repeating the STRUCTURE and PCoA in hierarchical analyses that excluded those divergent samples (as recommended by Janes et al., 2017; Pritchard & Wen, 2004). We measured the amount of genetic variation partitioned among geographic locations in each of the *Acropora* asp‐c and *I. brueggemanni* collections using *F*_ST_ with hierarchical AMOVA in GenAlEx. These analyses measured variation among the four archipelagos (*F*_RT_) of Ashmore Reef, the central Kimberley, Buccaneer Archipelago and Dampier Peninsula; among sites within archipelagos (*F*_SR_); and among all sites (*F*_ST_). We also calculated pairwise *F*_ST_ between all sites. We tested for statistical significance in all AMOVAs using 999 random permutations. Some *Acropora* asp‐c sites had small sample sizes, but because we employed thousands of SNPs, estimation of *F*_ST_ for samples sizes >4 (Willing, Dreyer, & van Oosterhout, 2012) and even >2 (Nazareno, Bemmels, Dick, & Lohmann, 2017) is likely to be robust. However, we also calculated an AMOVA for *Acropora* asp‐c lineage using only those sites where *n* ≥ 9.

### 2.5 Inter‐reef to within‐reef population structure

We investigated population genetic structure at the inter‐reef to within‐reef scale in the *Acropora* asp‐c lineage and *I. brueggemanni* using spatial autocorrelation analysis on the two archipelagos that were sampled in most detail in the inshore Kimberley (Dampier Peninsula and Buccaneer Archipelago). Spatial autocorrelation uses the spatial position and genetic identity of each individual. This analysis is therefore well‐suited to establishing the finest scale of genetic structure, is sensitive to recent dispersal processes and is robust to most natural characteristics of plant or animal populations (Double, Peakall, Beck, & Cockburn, 2005; Epperson, 2005). We calculated the autocorrelation between the genetic distance (codominant genotypic) and geographic (Euclidean) distance of all pairs of individuals that fell within a given distance class and plotted each autocorrelation coefficient, *r*, against its distance class in GenAlEx. Under conditions of restricted gene flow, *r* is expected to be positive and stable at short‐distance classes; then, a subsequent decline in *r* indicates the “genetic patch,” and the y‐intercept indicates a balance between genetic drift and gene flow before *r* becomes negative (Epperson & Li, 1996; Smouse & Peakall, 1999; Sokal & Wartenberg, 1983). Initial analysis of *I. brueggemanni* showed that the site Kooljaman (see Figure 1) was clearly separate from the general patterns of spatial genetic structure and so was excluded from this analysis. This decision also provided geographic consistency with the study of *A. aspera*. We tested for statistical significance of *r* at each distance class, by generating a 95% confidence interval about *r* via 1,000 bootstrap trials and drawing (with replacement) from within the set of pairwise comparisons for a specific distance class. We inferred significant spatial genetic structure when the confidence interval did not straddle *r* = 0.

We also estimated fine‐scale genetic structure with AMOVA among Bathurst, Pope and Mermaid Islands reefs (*F*_RT_REEFS_); between sites within these reefs (*F*_SR_SITES_); and among all these sites (*F*_ST_SITES_) for *I. brueggemanni*. This analysis was only possible in this species because we sampled replicate sites at these three reefs. We calculated pairwise *F*_ST_ between all sites and tested for statistical significance with 999 random permutations.

### 2.6 Environment, genetic structure and diversity

We quantified the effect of the environment on the population genetic structure and diversity of the *A*. asp‐c lineage and *I. brueggemanni* using the Bayesian method implemented in GESTE (Foll & Gaggiotti, 2006). Specifically, we tested whether environmental heterogeneity was associated with variation in levels of genetic differentiation and diversity. GESTE calculates posterior probabilities with a generalized linear model to identify the most important environmental influences on site‐specific levels of genetic differentiation or local *F*_ST_. Local *F*_ST_ is the mean distance between each focal population and all other population samples and provides a measure of genetic distinctiveness of each local population relative to the entire metapopulation. This approach is node‐based and accounts for the nonindependence inherent in multiple pairwise comparisons (Foll & Gaggiotti, 2006; Riginos, Crandall, Liggins, Bongaerts, & Treml, 2016). We analysed only those sites where *n* ≥ 9 to account for small sample sizes at some sites in the *Acropora* asp‐c lineage. This meant that the genetic and geographic outlying site of Ashmore Reef was excluded. For both corals, we used a sample size of 10,000 and a thinning interval of 50 (total of 5 x 10^5^ iterations), 10 pilot runs with a length of 5,000 and an additional burn‐in of 50,000. We included six environmental factors: latitude, longitude, the 90th percentile in tidal height (m), range in sea surface salinity (PSS), range in sea surface temperature (°C), dissolved oxygen (mL/L), nitrate (μmol/L) and water clarity (the diffuse attenuation coefficient at 490 nm/m). The latter four factors were assembled from several sources of remotely sensed and in situ measured oceanographic data specific to our sites. Tidal height was sourced from model output of the Renewable Energy Atlas of Australia (David Griffin, CSIRO Oceans and Atmosphere, pers. comm., available at http://www.marine.csiro.au/~griffin/ORE/data/). Sea surface salinity and temperature were sourced from the MARSPEC ecological archives (Sbrocco & Barber, 2013) at a 30‐arc‐second spatial resolution collected from 2002 to 2010. Water clarity was sourced from the Bio‐ORACLE data set (http://www.bio‐oracle.org/) at 5‐arc‐minute resolution one kilometre offshore of the sites and collected from 2002 to 2009. The environmental factors were normalized and transformed into the mean absolute difference between values at the focal population and all the other sampled populations.

We also investigated the seascape influences on the diversification of lineages within *A. aspera* by testing whether sites with greater environmental heterogeneity were associated with greater genetic diversity in these corals. To this end, we used a simple linear regression to correlate gene diversity (unbiased expected heterozygosity) with the same environmental factors used in the GESTE analysis at each site in the *Acropora* asp‐c lineage.

## 3 RESULTS

### 3.1 Cryptic diversity in *Acropora aspera*

**FIGURE 2:**
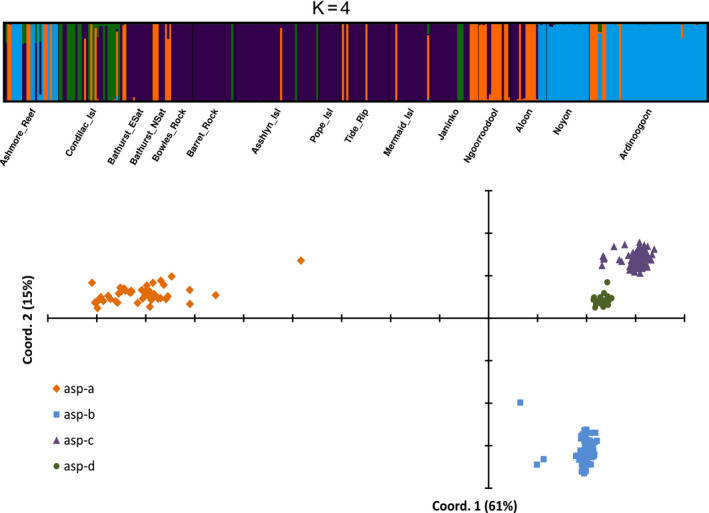
Clustering analysis results from the entire *Acropora aspera* collection. Upper panel shows the bar plot of membership coefficients of individual corals calculated with STRUCTURE v2.3 with no prior information for *K* = 4. CLUMPAK calculated this plot from 10/10 runs and a similarity score = 0.999 and mean (LnProb) = −53747.610. Lower panel shows principal coordinate analysis calculated from individual pairwise genotypic distance. Individuals are colour‐coded according to the clusters assigned by the STRUCTURE analysis. Percentage of variation explained by each axis is given in brackets

The cluster analysis of 329 unique genotypes (genets) of *A. aspera* revealed four sympatric genetic lineages (hereafter referred to as *Acropora* asp‐a, asp‐b, asp‐c and asp‐d). STRUCTURE indicated ΔK was the highest at *K* = 4 (Figure D1), and membership coefficients (*q*) were very strong (*q* > 0.90; Figure 2) for most individuals across these four clusters using the stringent data set of 585 loci. Although the Ln(Pr(X|*K*) plot indicated the presence of additional finer level of structure with optimal *K* = 8, *q*‐values were much weaker at this *K* and likely reflect population geographic structure within lineages. Further, the PCoA supported the Δ*K* results, distinguishing four discrete lineages within the entire collection of *A. aspera*, two of which (asp‐c and asp‐d) were relatively closely related (Figure 2). There were major differences among these lineages across the genome, with private alleles in one of the four lineages at 247 loci (P*_A_*_ asp‐a_ = 98, P*_A asp‐b_* = 64, P*_A asp‐c_* = 69 and P*_A asp‐d_* = 26). Pairwise *F*_ST_ between lineages was very large, ranging from 0.480 to 0.704 (Table 3). These estimates of divergence were highly congruent with the relaxed data set of 3,698 loci (Table D1). Further, comparison of different axes of PCoA shows that segregation of clusters, including asp‐c and asp‐d, was strong in multidimensional space (Figure D2).

**TABLE 3:** Pairwise *F*_ST_ values among lineages of corals from the entire *Acropora aspera* collection identified with STRUCTURE from 585 loci

	asp‐a	asp‐b	asp‐c	asp‐d
asp‐a	0.000			
asp‐b	0.643	0.000		
asp‐c	0.704	0.498	0.000	
asp‐d	0.643	0.484	0.480	0.000

A weak geographic pattern was evident in the distribution of the four *A. aspera* lineages (Figure 2). The island sites of the Buccaneer Archipelago were mostly comprised of *Acropora* asp‐c. The mainland sites on the Dampier Peninsula were mostly *Acropora* asp‐b. Sites in the central Kimberley were mostly *Acropora* asp‐d. In contrast, both the *Acropora* asp‐c and *Acropora* asp‐a lineages were widely spread throughout all reefs and archipelagos sampled, but *Acropora* asp‐c was by far the most abundant.

Despite this weak geographic pattern, multiple lineages occurred at almost all sites. For example, all four lineages occurred at the central Kimberley site of Condillac Island, while Ashmore Reef was comprised of *Acropora* asp‐a, asp‐b and asp‐c. Crucially, the most closely related lineages (asp‐c and asp‐d) occurred side by side at many sites but exhibited pairwise *F*_ST_ of 0.480 (Table 3), indicating strong genetic isolation even when living in sympatry. Morphological assessments in the field, along with preliminary assessments of skeletal material, showed no clear macro‐morphological differences among the lineages (Figure D3). Gene diversity within each lineage also varied greatly and was highest in *Acropora* asp‐a (0.108) and lowest in *Acropora* asp‐d (0.054; Figure D4).

### 3.2 Inter‐archipelago to inter‐reef population structure

**FIGURE 3:**
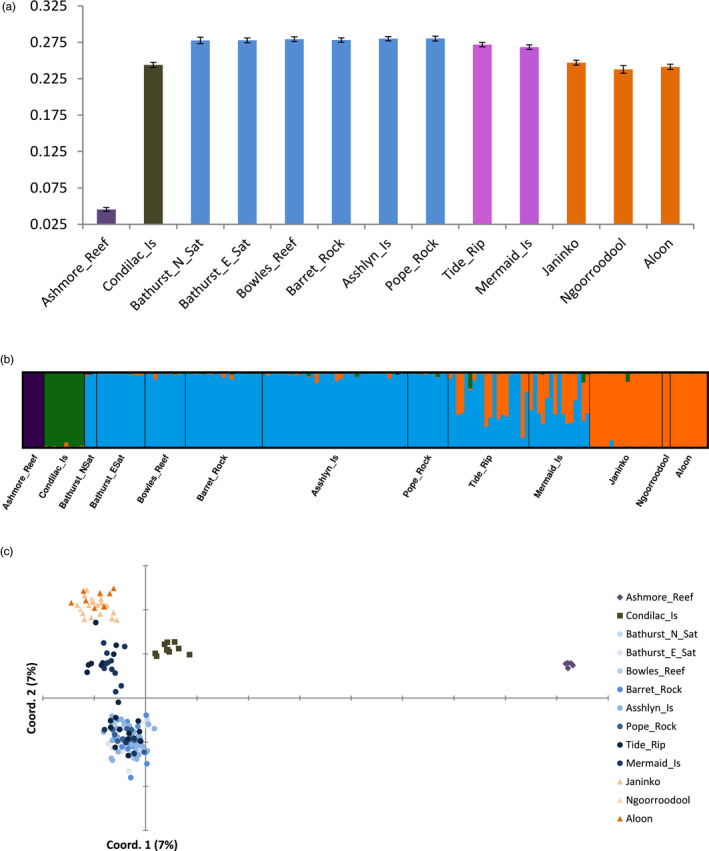
Distribution of genetic diversity through north‐west Australia in the *Acropora* asp‐c lineage. Panel A shows gene diversity at each site based on unbiased expected heterozygosity (± standard errors and trend line with *r* ^2^ value). Panel B shows the bar plot of membership coefficients of individual corals calculated in STRUCTURE v2.3 for *K* = 4. This plot is of the major mode produced by CLUMPAK calculated from 6/10 runs and a similarity score = 0.986, and a mean LnProb = −423687.042. The minor mode was almost identical. Panel C shows the principal coordinate analysis calculated from individual pairwise genotypic distance (percentage of variation explained by each axis is given in brackets)

**FIGURE 4:**
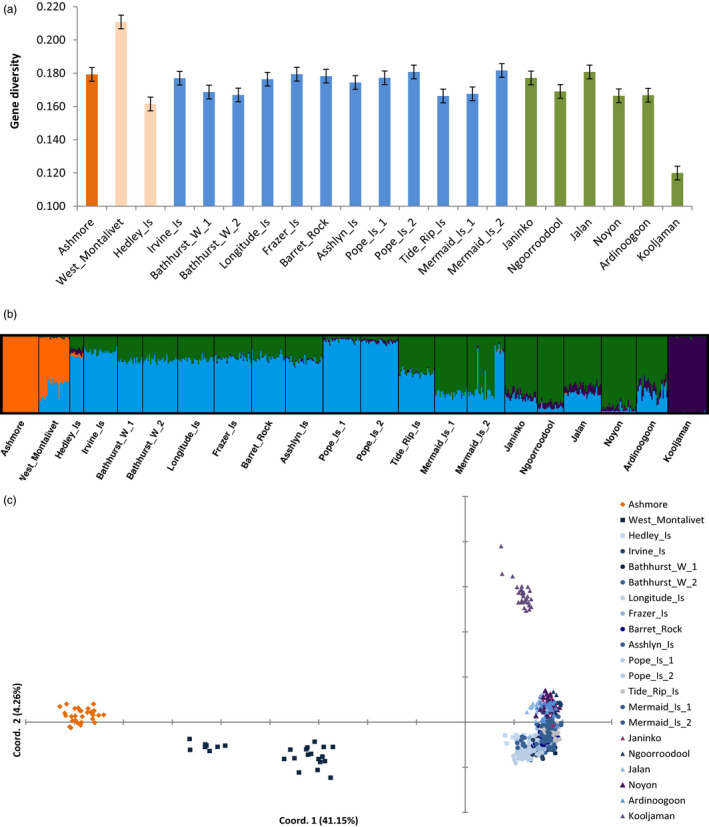
Distribution of genetic diversity throughout north‐west Australia in *Isopora brueggemanni*. Panel A shows gene diversity at each site based on unbiased expected heterozygosity (± standard errors and trendline shown). Panel B shows the bar plot of membership coefficients of individual coral calculated in STRUCTURE v2.3 for *K* = 4. This is the major mode plot produced by CLUMPAK calculated from 10/10 runs, and a similarity score = 0.985 and mean LnProb = −682167.990. Panel C shows the principal coordinate analysis calculated from individual pairwise genotypic distance (percentage of variation explained by each axis is given in brackets)

We focused on the A*cropora* asp‐c lineage using 2,894 loci for subsequent population‐level analysis in the *Acropora* data set. After removal of clones (final *n* = 169; Table 1 and Appendix C), average observed heterozygosity across all loci was 0.202, average expected heterozygosity was 0.247, and average *F*_IS_ was 0.122. These results suggested a general deficiency in heterozygotes expected under Hardy–Weinberg equilibrium. This result is very common in hard corals, especially in broadcast spawners (Ayre & Hughes, 2000; Mackenzie, Munday, Willis, Miller, & Van Oppen, 2004; Nishikawa & Sakai, 2005; Underwood, 2009; Underwood, Smith, van Oppen, & Gilmour, 2009; Whitaker, 2004), and indicate Wahlund effects brought about by nonrandom mating within sites due to spatial and/or temporal admixture. Gene diversity (unbiased expected heterozygosity at each site) was higher in the centre of the sampling area at the Buccaneer Archipelago sites than at the Dampier Peninsula or the central Kimberley and was very low at Ashmore Reef (Figure 3a). After removal of clones (*n* = 561; Table 2 and Appendix C), average observed heterozygosity of *I. brueggemanni* was 0.176, average expected heterozygosity was 0.173, and average *F*_IS_ was −0.069 across all loci, with very few loci in genotypic disequilibrium in this species. Gene diversity of *I. brueggemanni* was relatively constant over most of the sampling sites with two exceptions: West Montalivet in the far east was the highest (*H*_E_ = 0.211), and Kooljaman in the far west was the lowest (*H*_E_ = 0.119; Figure 4a).

The *Acropora* asp‐c lineage segregated according to four geographic locations in all cluster analyses. The STRUCTURE results revealed that optimal *K* = 3 with ΔK method and *K* = 4 with the Ln (Pr(X|K) method (Appendix D, Figure D5). At *K* = 4, *q* was greater than 90%, separating membership to Ashmore Reef, the central Kimberley site (Condillac Island), the Buccaneer Archipelago (Bathurst E Satellite, Bathurst N Satellite, Bowles Reef, Barret Rock, Asshlyn Islands and Pope Island) or the Dampier Peninsula (Janinko, Ngoorroodool and Aloon; Figure 3b). Although half the corals at Tide Rip and Mermaid islands exhibited strong affinities to the Buccaneer Archipelago cluster (*q* > 85%), the remainder exhibited intermediate ancestry (*q* ~ 0.50) between the Buccaneer and Dampier Peninsula clusters (Figure 3b). This geographic segregation into four clusters and the patterns of admixture were well supported by the PCoA (Figure 3c). Most of the geographic variation within the *Acropora* asp‐c lineage was attributed to differences among the four archipelagos in the AMOVA (*F*_RT_ = 0.094, *p* < .001). However, small and significant differences were detected among sites within archipelagos (*F*_SR_ = 0.008, *p* < .05). Consequently, overall subdivision among all sites was moderate but highly significant (*F*_ST_ = 0.101, *p* < .001). The largest pairwise differences were between the Ashmore site and all the other sites, with average *F*_ST_ of 0.380 (±SE 0.011; Table D2). Levels of subdivision were therefore weaker when Ashmore and other sites with sample sizes ≤ 8 were excluded in the AMOVA, but overall patterns and statistical significance were the same (Table D3).

The geographic structuring observed across a range of spatial scales in *Acropora* asp‐c was far more pronounced in *I. brueggemanni*. Utilizing 2,125 loci, STRUCTURE analysis revealed maximum ∆*K* was at K = 2, with very strong membership (*q* = 1) of all corals to either an offshore Ashmore cluster or an inshore cluster (except for West Montalivet that had *q ~* 50% to both clusters). However, at *K* > 2, clusters continued to segregate according to geography, and the Ln (Pr(X|K) method identified optimal *K* = 10. An additional cluster was formed by Kooljaman at K = 3 (Appendix E, Figure E1) and by the Dampier Peninsula at *K* = 4 (Figure 4b). At *K* = 4, sites at Tide Rip and Mermaid islands exhibited admixed membership to the Dampier and the Buccaneer clusters, either within individuals (at Tide Rip Island, *q* ~ 50% for all individuals) or among individuals (at Mermaid Island_2, *q* > 75% to either the Dampier or Buccaneer cluster; Figure 4b). At *K* = 5, a cluster at Pope Island segregated. At *K* = 6, the sites of Irvine and Bathurst West segregated. At *K* > 6, the corals from West Montalivet segregated from the Ashmore cluster (Figure E1). This geographic segregation was well supported by the PCoA at the inter‐archipelago scale (Figure 4c), as were patterns of admixture between Buccaneer Archipelago and Dampier Peninsula samples at the inter‐reef scale (Figure E2). Strong geographic structure in *I. brueggemanni* across multiple scales was also detected by the AMOVA. Large and significant variation was attributed to differences among the four archipelagos (*F*_RT_ = 0.151, *p* < .001) and among sites within archipelagos (*F*_SR_ = 0.092, *p* < .001), yielding a large overall level subdivision among all sites (*F*_ST_ = 0.230, *p* < .001). Pairwise *F*_ST_ between Ashmore Reef and the inshore reefs was very high and averaged 0.450 (±SE 0.015), but was lowest with West Montalivet (*F*_ST_ = 0.227; Table E1). Pairwise *F*_ST_ was also notably high between Kooljaman and the other inshore sites (*F*_ST_ = 0.241 ± SE 0.019).

### 3.3 Inter‐reef to within‐reef population structure

**FIGURE 5:**
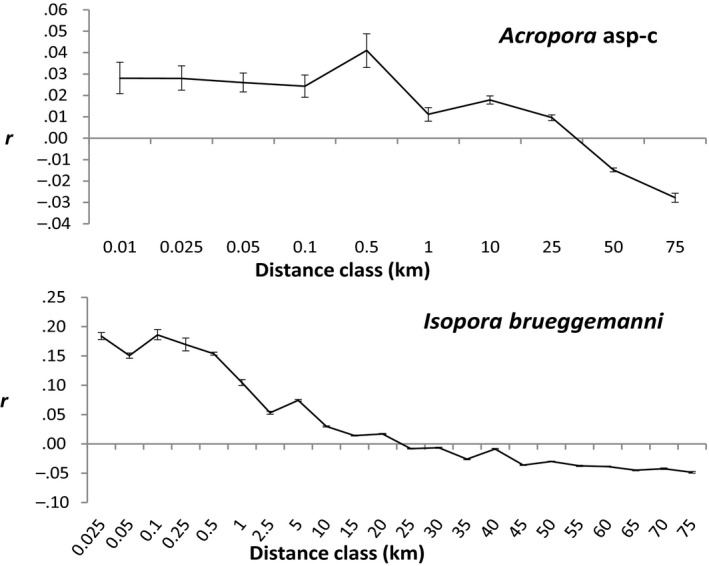
Spatial autocorrelation analyses of the genetic correlation coefficient (*r*) as a function of distance for the *Acropora* asp‐c lineage (upper panel) and the *I. brueggemanni* (lower panel) corals sampled from the Dampier Peninsula and the Buccaneer Archipelago. The bootstrapped 95% confidence intervals were generated by 1,000 bootstrap trials. X‐axes differ slightly because of more extensive spatial sampling of sites in *I. brueggemanni*

There was significant genetic structure over fine scales within the Dampier Peninsula and Buccaneer Archipelago in both *Acropora* asp‐c and *I. brueggemanni*. The positive correlation coefficient (*r*) in both plots was significant and relatively constant up to 500 m (Figure 5). After this initial plateau, *r* declined, reflecting size of the genetic patch. The correlation coefficients became negative at 35 km for *Acropora* asp‐c and 20 km for *I. brueggemanni*, showing the limits to the homogenizing influence of gene flow as the primary determinant of genetic composition.

The AMOVA of *I. brueggemanni* collections that included replicate sites within reefs at Bathurst, Pope and Mermaid islands showed significant subdivision between sites within reefs (*F*_SR_SITES_ = 0.010, *p* ≤ .01), over distances of approximately 500m. Pairwise *F*_ST_ comparisons indicated the significant differences between sites occurred at reefs of Pope Island (*F*_ST_ = 0.013, *p* ≤ .030) and Mermaid Island (*F*_ST_ = 0.014, *p* ≤ .020), but not Bathurst West (*F*_ST_ = 0.002, *p* ≤ .226). Despite this subdivision within reefs, much more of the variation was attributed by AMOVA to subdivision among the three reefs (*F*_RT_REEFS_ = 0.085, *p* ≤ .001).

### 3.4 Environment, genetic structure and diversity

There was a strong association between environment and genetic structure and diversity in both corals. The GESTE analysis revealed that tide formed the highest probability model for *Acropora* asp‐c (*p* = .556) and for *I. brueggemanni* (*p* = .432; Table 4). All other models exhibited much lower probabilities (*p* ≤ .1). The slope of regression was negative for both corals (Table 4), showing that site‐specific genetic differentiation (local *F*_ST_) decreased with increasing tidal magnitude. However, the deviation from the regression was moderate for both corals (Table 4), suggesting other untested environmental factors also contributed to the genetic patterns.

**TABLE 4:** Results of GESTE analysis for *Acropora* asp‐c and *I. brueggemanni* showing mean regression coefficients (α) for each factor that was retained in the best model. Also given is mode of deviation from regression of best model (ơ^2^)

species	factor	mean/mode	95% HPDI
*Aspera* asp‐c			
α0	Constant	−3.590	[−4.080; −3.110]
α1	Tidal height	−0.981	[−1.480; −0.496]
ơ^2^		0.402	[0.175; 1.25]
*I. brueggemanni*			
α0	Constant	−2.050	[−2.233; −1.740]
α1	Tidal height	−0.632	[−0.924; −0.338]
ơ^2^		0.373	[0.226; 0.793]

**FIGURE 6:**
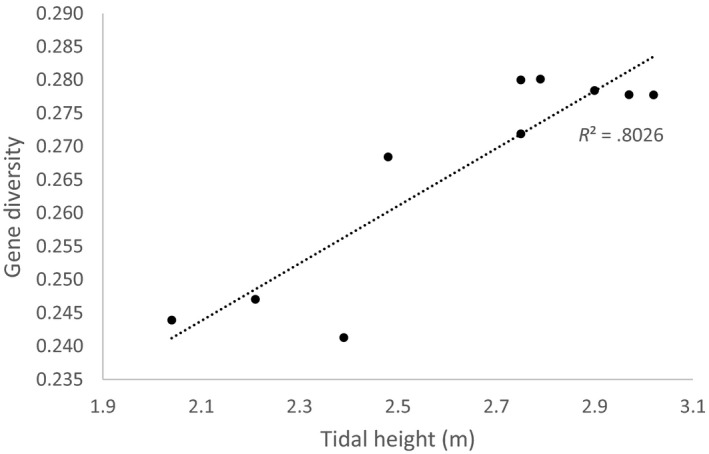
The correlation between tidal height and gene diversity (unbiased expected heterozygosity) of the *A. aspera* asp‐c lineage from the inshore Kimberley for sites with *n* ≥ 9

Tidal magnitude also exhibited a strong association with gene diversity at each site within the *Acropora* asp‐c lineage. Specifically, sites with bigger tides exhibited greater diversity (R^2^ = 0.806, Figure 6). We observed a weaker positive relationship between range in sea surface temperature and gene diversity (R^2^ = 0.335), and no relationship with the other environment factors.

## 4 DISCUSSION

Strong genetic divergence and restricted population connectivity characterized the distribution of genetic diversity in two reef‐building corals from north‐west Australia. These characteristics were evident across a wide range of spatial scales in both the spawning coral, *Acropora aspera*, and the brooding coral, *Isopora brueggemanni*. This consistency between species with different life histories indicated that the heterogeneous seascape and powerful oceanographic currents of this region have important influences on their metapopulation dynamics. Environmental influences not only promote strong genetic differentiation between bioregions and archipelagos (tens to hundreds of kilometres) and regular local recruitment within reefs (tens of metres to a few kilometres), but also rare longer‐distance connectivity between reefs within archipelagos (kilometres to tens of kilometres). Underlying this spatial genetic structure, we discovered several highly divergent and cryptic lineages in *A. aspera* that co‐occur on the same reef patch.

### 4.1 Cryptic diversity and Kimberley corals

We detected four distinct genetic lineages in *A. aspera* that were not distinguished by macro‐morphological characteristics. Pairwise *F*_ST_ between the four lineages was large (*F*_ST_ ≥ 0.469), and private alleles were observed at more than half the loci analysed. These results indicate inter‐breeding between lineages is rare (sensu Moritz, 2002), despite often co‐occurring on the same reef patch. This result is consistent with many genetic studies in other regions that have detected cryptic diversity in *Acropora* (e.g. Ladner & Palumbi, 2012; Ohki, Kowalski, Kitanobo, & Morita, 2015; Sheets et al., 2018; Wallace & Willis, 1994) as well as other scleractinian (e.g. Forsman, Barshis, Hunter, & Toonen, 2009; Miller & Babcock, 1997; Pinzon et al., 2013). The detection of cryptic lineages is also consistent with extensive evidence throughout the Kimberley and north‐west Australia of high inter‐specific‐level genetic diversity within reef‐building coral species (Richards et al., 2016; Richards, Miller, Miller, & Wallace, 2013; Rosser, 2015, 2016; Thomas et al., 2014; Underwood et al., 2018).

Our *A. aspera* data also illuminate evolutionary forces unique to the coral reefs of north‐west Australia. A high level of SNP diversity in north‐west Australia contrasts to that observed from sequences of the ribosomal DNA ITS region, in which *A. aspera* was the only example out of five sister species on the Great Barrier Reef in eastern Australia that did not exhibit strong genetic divergence within the morphospecies (Van Oppen, Willis, Van Rheede, & Miller, 2002). Further, our analysis showed that greater gene diversity in the *Acropora* asp‐c lineage occurred at sites with bigger tides. Considered in the context of the recent (<8,000 years) history of coral reefs of the inshore Kimberly (Solihuddin et al., 2016; Wilson, 2013), this result suggests the heterogenous seascape of the Kimberley may have led to the rapid evolution of unique and often cryptic coral diversity.

Although many mechanisms are likely involved in *evolution* of the distinct lineages detected here in *A. aspera*, timing of spawning (prezygotic barrier) is the best explanation for the *maintenance* of reproductive isolation among sympatric lineages. Indeed, direct evidence of species‐level genetic differences has been observed in *Acropora* lineages that appear identical but spawn in either spring or autumn (Gilmour et al., 2016; Rosser, 2016; Rosser et al., 2020) or in different months of the same season (Dai, Fan, & Yu, 2000; Ohki et al., 2015; Wolstenholme, 2004). A revision of the taxonomic status of *A. aspera* that integrates genetic, micro‐morphological and reproductive data is warranted.

### 4.2 Population structure and connectivity

The overall level of genetic subdivision among reefs within the *Acropora* asp‐c lineage (*F*_ST_ = 0.101) was half that of the brooder *I. brueggemanni* (*F*_ST_ = 0.230). This result reflects the potential for greater dispersal by spawned larvae through longer precompetency periods compared with brooded larvae (see Appendix A) and is also consistent with results from a similar comparison of species with different life histories at the offshore reefs of north‐west Australia (Thomas et al., 2020). However, genetic differentiation was consistently correlated with geographic distance at all scales studied, despite differences in magnitude between species.

At the broadest scale, the largest genetic divergence occurred between offshore and inshore bioregions in both species. This result supports the absence of cross‐shelf connectivity in other genetic (Underwood, 2009; Underwood et al., 2018), oceanographic (D'Adamo et al., 2009) and biodiversity studies (Richards, Bryce, & Bryce, 2018; Wilson, 2013). Also at broad scales, three distinct genetic groups were observed among the inshore reefs, with clusters segregating the central Kimberley, the Buccaneer Archipelago and the Dampier Peninsula in both species.

At an intermediate scale, we detected positive spatial structure for colonies separated up to 20 km for *I. brueggemanni* and 35 km for *A. aspera*. The positive structure over distances of a few tens of kilometres reflects the distance over which dispersal is rare enough that the diversifying effects of genetic drift counter the homogenizing influence of gene flow. Such positive structure was apparent even when we analysed the Dampier Peninsula and Buccaneer Archipelagos separately, showing that results were not greatly influenced by inter‐archipelago differentiation (data not shown). This result is consistent with offshore studies in north‐west Australia from other brooding species (Thomas et al., 2020; Underwood et al., 2009, 2018; Underwood, Smith, van Oppen, & Gilmour, 2007) but contrasts to recent evidence of panmixia over these local spatial scales (<100 km) in a different broadcast spawning coral (*Acropora digitifera*; Thomas et al., 2020). Therefore, the evidence gathered to date suggests many coral populations that are separated by more than a few tens of kilometres are demographically independent, but the environmental heterogeneity of the inshore Kimberley may further restrict connectivity in spawners.

At a local scale, colonies of both species less than 500 m apart were more closely related than more distance colonies. This distance indicates the genetic patch of complete mixing. The size and distinctness of the genetic patch are likely due to fine‐scale environmental heterogeneity that influences survival after settlement (e.g. Johnson & Black, 1982). However, we also suspect life histories play an important role. Larvae of brooders can settle soon after release and recruit very close to their parents. Here, significant differentiation was observed in *I. brueggemanni* between colonies and sites on the same reef, and the positive autocorrelation was much higher than for the spawner. In contrast, larvae of broadcast spawners spend at least a few days in the plankton. This means the fine‐scale genetic patchiness of *A. aspera* also likely reflects the influence of sticky water and tidally driven eddies that concentrate larvae together and limit mixing of a wider larval pool (Andutta, Kingsford, & Wolanski, 2012; Selkoe et al., 2010; Wolanski & Spagnol, 2000).

### 4.3 Management implications

Kimberley corals thrive in extreme conditions with especially wide ranges in temperature, irradiance and water quality (Wilson, 2013). However, even in the Kimberley, bleaching occurs when anomalous heatwaves exceed those tolerances (Gilmour et al., 2019; Hughes et al., 2017; Schoepf et al., 2015). Recovery after such disturbances requires the continued production of demographically important numbers of recruits from local populations over small spatiotemporal scales. In addition, persistence of the metapopulation as a whole requires connectivity networks that enable rarer but evolutionarily important dispersal over broader scales (Gaggiotti, 2017). Such networks maintain the standing genetic diversity and enhance resilience through the spread of adaptive alleles among local populations as the environment changes (van Oppen & Gates, 2006; Torda et al., 2017). Networks of marine reserves are the primary spatial tool for protecting connectivity in habitat‐forming species that are vulnerable to climate change. This study addresses specific research priorities identified by Kendrick et al. (2016) required to inform management of the five‐million‐hectare Great Kimberley Marine Park by empirically assessing population connectivity and genetic diversity of reef‐building corals in this region.

We found no evidence of contemporary cross‐shelf connectivity, so inshore reefs rely on their own stocks not only to supply recruits every generation, but also for genetic diversity to adapt to climate change over multiple generations. These inshore populations are maintained by locally produced recruits at the scale of reef or reef patch, with very few brooded or spawned larvae dispersing and surviving more than 35 km from place of origin. However, our seascape analysis also revealed that genetic structure and diversity were strongly associated with tidal magnitude; sites with bigger tides were more connected to the entire metapopulation (in both species) and were more diverse (in *Acropora* asp‐c). Therefore, strong tidally driven currents appear to have increased the likelihood of occasional larval dispersal between local populations. Conversely, the deep‐water tidal current at Sunday Strait appears to be a semi‐permeable barrier to dispersal of larvae between the genetically distinct Dampier Peninsula and Buccaneer Archipelago. This result is consistent with other studies that have shown strong oceanic currents often act as “leaky” barriers to larval dispersal that override the influence of biological factors on genetic structure such as planktonic period (Baums, Paris, & Cherubin, 2006; Hohenlohe, 2004; Suzuki et al., 2016).

The congruent patterns among two species with very different modes of reproduction suggest that a single spatial marine management strategy may be used to aid resilience of all coral populations in this region. We recommend that multiple sanctuary networks be spaced at distances no greater than a few tens of kilometres. More specifically, the Dampier Peninsula and Buccaneer Archipelago should be managed as demographically independent systems that sustain their populations through production of local recruits. Further, the population at Kooljaman was the most genetically divergent and depauperate of the *I. brueggemanni* sites and is probably small, isolated and close to the limits of its south‐western range. Therefore, Kooljaman may be more vulnerable to local extinction compared with other reefs studied here. Lastly, the genetic signatures at Tide Rip and Mermaid islands were admixed between the Dampier Peninsula and Buccaneer Archipelago, indicating these reefs provide stepping stones for occasional genetic exchange between the archipelagos important for the adaptive capacity of the metapopulation. We suggest these islands should be considered conservation priorities.

The discovery of cryptic lineages within *A. aspera* also has implications for management. Such unrecognized diversity is probably common in these systems (Richards et al., 2016), and biodiversity estimates need to account for this (Fišer, Robinson, & Malard, 2017). Recent evidence indicates ecosystem productivity increases with species richness in many wild populations (Duffy et al., 2017), and coral biodiversity enhances reef ecosystem function (Clements & Hay, 2019). Therefore, the discovery of unrecognized inter‐specific diversity may well confer greater resilience to changing environment. Alternatively, if inter‐breeding among lineages is rare, their effective population sizes will be smaller than expected, increasing their susceptibility to Allee effects and reproductive failure following reductions in density after disturbance (Knowlton, 2001). In addition, the relatively low genetic diversity of less abundant lineages such as *Acropora* asp‐d may reflect a limited adaptive capacity. Such lineages are likely vulnerable to silent extinction.

Coral reefs worldwide are threatened by the increased frequency and severity of marine heatwaves (Hughes et al., 2018; Van Hooidonk, Maynard, & Planes, 2013). The impacts of such temperature anomalies appear to override the well‐documented ecological benefits of no‐take reserves (Graham et al., 2020). Therefore, the ecosystem trajectory of most coral reefs will primarily depend on the rate at which carbon emissions are reduced (Hughes et al., 2017). Nevertheless, conservation strategies that sustain existing connectivity networks will be important (van Oppen & Gates, 2006). Such strategies that protect reefs from local pressures will promote demographic recovery in the short term by capitalizing on the natural variation in resilience to heatwaves of local populations and also the adaptive capacity of coral metapopulations in the longer term. This study illuminates the hidden genetic structuring of two key species of habitat‐forming corals to support such local management actions.

## CONFLICT OF INTEREST

None declared.

## Supporting information

Appendix A

Appendix B

Appendix C

Appendix D

Appendix E

## Data Availability

Data for genotypes, SNP summary statistics and coral coordinates are available via Dryad Digital Data Repository given at https://doi.org/10.5061/dryad.w9ghx3fm3. Dryad submission includes:
**aspera_DArT_Report_34304_SNPs**: Excel file with two‐row data from 34,304 SNPs called for all *Acropora aspera* samples. Includes all ramets and information of each locus provided by DArT propriety pipeline.
**bruegs_DArT_Report_23165_SNPs**: Excel file with two‐row data from 23,165 SNPs called for all *Isopora brueggemanni* samples. Includes information of each locus provided by DArT propriety pipeline.
**aspera_all_Kimberley_585_loci.**: Excel file in GenAlEx format for 585 SNPs used to delineate clusters in the entire *Acropora aspera* data set. Second worksheet includes the summary statistics for each locus for each lineage.
**aspera‐c_Kimberley_2898_loci**: Excel file in GenAlEx format for 2,898 SNPs used for the population genetics analysis of Aspera‐c lineage of 169 genets. Includes georeferenced coordinates for location of each genet. Second worksheet describes the summary statistics for each locus for site and averaged across sites.
**bruegs_Kimberley_2132_loci**: Excel file in GenAlEx format for 2,132 SNPs used for population genetics analysis of 561 *Isopora brueggemanni* genets. Includes georeferenced coordinates for location of each genet. Second worksheet describes the summary statistics for each locus for site and averaged across sites.
**Metadata** for this study is given at: http://catalogue.aodn.org.au/geonetwork/srv/eng/metadata.show?uuid=fb1d80bf‐6ef2‐4150‐9479‐22b4240435a7
