# Supplementary material for: Extreme seascape drives local recruitment and genetic divergence in brooding and spawning corals in remote north‐west Australia
Source: Evol Appl. 2020 Jun 22;13(9):2404–21. doi: 10.1111/eva.13033 (PMC7513722; doi:10.1111/eva.13033)
Supplement: Supplementary file 1 — Appendix A [file EVA-13-2404-s001.docx]

# Appendix A: Reproduction, ecology and dispersal capacity of *Acropora aspera* and *Isopora brueggemanni*

In Western Australia, *Acropora aspera* occurs from the tropical oceanic shoals in the north to the subtropical Abrolhos Islands in the south (Veron & Marsh, 1988). It reaches high abundance at inshore reefs in the Kimberley where it occurs on exposed intertidal reef flats (Richards et al., 2015). *Acropora aspera* is a broadcast spawner, whereby eggs and sperm are released during mass spawning events, after which fertilisation and larval development occur in the plankton. Data on spawning time of *A. aspera* in the Kimberley is sparse, but a primary spawning most likely occurs in the Austral autumn, and there may be a secondary spawning in spring (Gilmour et al., 2016). In contrast to brooded larvae, broadcast spawned larvae must spend a few days in the plankton before they are competent to settle, and thus have stronger potential for dispersal away from natal reef compared with brooding corals. However, if suitable substrate is available, the majority of larvae probably settle as soon as they are competent, as their probability of settling and surviving drops rapidly the longer they spend in the plankton (Baird, 2004; Harrison & Wallace, 1990). Vegetative fragmentation may well supplement the broadcast spawning reproduction particularly in exposed, reef platform habitats. *A. aspera* is considered to be relatively easily identified in the field although it can be confused with *Acropora pulchra*. *A. aspera* is listed as “vulnerable” on the IUCN Red List of Threatened Species (Aeby et al., 2014).


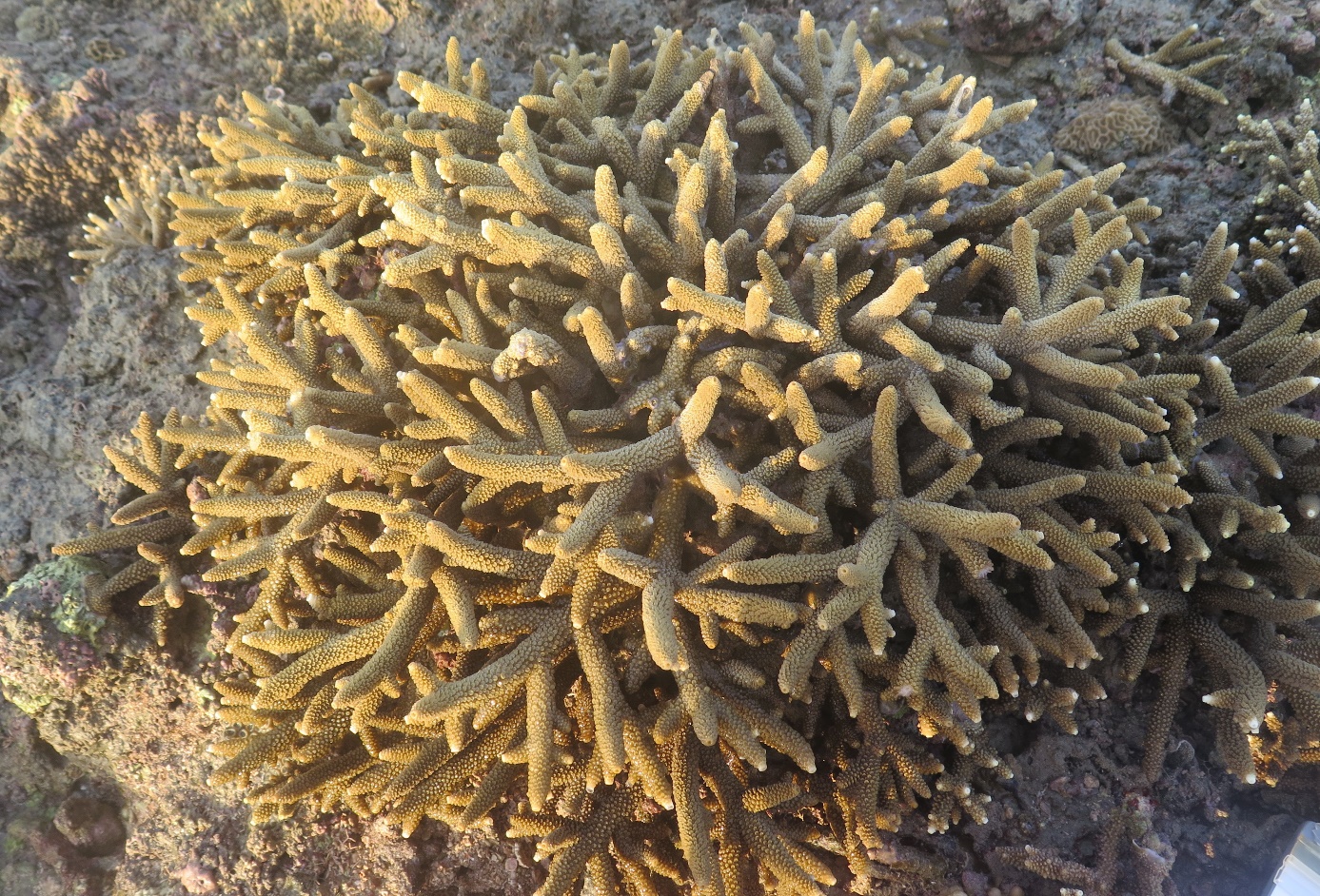


Figure A1 Photo of *Acropora aspera* from the intertidal inshore Kimberley by Zoe Richards

*Isopora brueggemanni* is one of the most easily identifiable corals within the family Acroporiidae due to its slender branches and multiple axial corallites (Wallace, 1999). In the Kimberley, it occurs in shallow (< 20 m) water, especially on exposed upper reef slopes and sand flats, and is abundant and widespread on inshore and offshore reefs (Richards et al., 2014). Unlike *A. aspera*, *I. brueggemanni’ s* sexual reproductive mode involves release of sperm into the water column and fertilisation of eggs within the polyp (although self-fertilisation has also been reported Okubo et al., 2007). The resulting larvae are brooded within the polyp and then released at an advanced developmental stage. Planula release is likely to be extended over several months through spring to autumn in the Kimberley (Gilmour et al., 2016), but the exact timing is not currently known in this region. Larvae are probably capable of settling within hours of release (minimum competency period) (Harrison & Wallace, 1990). Brooding hard corals are generally characterised by strong levels of genetic subdivision, and self-seeding is well established (Ayre & Dufty, 1994; Ayre & Hughes, 2000; Underwood et al., 2007, 2009; van Oppen et al., 2011 ). Recent work on I*. brueggemanni* offshore reefs of northwest Australia revealed extensive self recruitment and restricted connectivity between habitats (Thomas et al., 2020). However, brooded planulae are relatively large when released into the water column and some contain maternal zooxanthellae, and therefore appear to be provisioned for a long maximum competency period (Harrison & Wallace, 1990). Thus, dispersal is likely to be bimodal, with long-distance dispersal (teleplanic) occasionally supplementing more routine, philopatric dispersal or self-seeding. The success of either strategy is likely to depend on environmental and demographic conditions: stable healthy populations are expected to be maintained by locally derived recruits, while recovery after sever disturbance is expected to be initiated from input of exogenous larvae followed by local recruitment (Underwood et al., 2007; Underwood et al., 2018). Similar to *A. aspera*, sexual reproduction can also be supplemented asexual vegetative fragmentation, which is likely to be more common in exposed, platform habitats. *I. brueggemanni* is listed as “vulnerable” on the IUCN Red List **
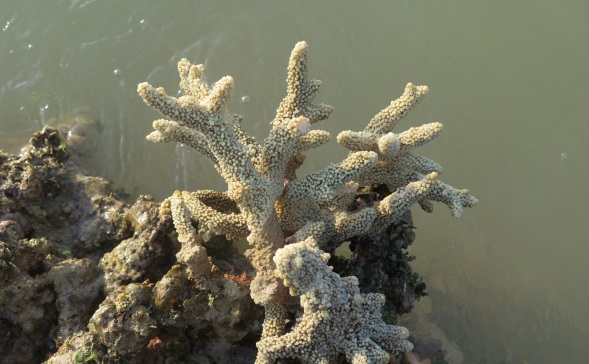
**of Threatened Species (Richards et al., 2008).

Figure A2 Photo of *Isopora brueggemanni* from the intertidal inshore Kimberley by Jim Underwood

### References

Aeby, G., Delbeek, J. T., Lovell, E. R., Richards, Z. T., Reboton, C., & Bass, D. (2014). Acropora aspera. The IUCN Red List of Threatened Species 2014: e.T133132A54200688. doi:10.2305/IUCN.UK.2014-1.RLTS.T133132A54200688.en

Ayre, D. J., & Dufty, S. (1994). Evidence for restricted gene flow in the viviparous coral *Seriatopora hystrix* on Australia's Great Barrier Reef. Evolution*,* 48, 1183-1201.

Ayre, D. J., & Hughes, T. P. (2000). Genotypic diversity and gene flow in brooding and spawning corals along the Great Barrier Reef, Australia. Evolution*,* 54, 1590-1605.

Baird, A. H. (2004). The ecology of coral larvae: settlement patterns, habitat selection and the length of the larval phase. Journal & Proceedings of the Royal Society of New South Wales*,* 137, 43.

Gilmour, J., Speed, C. W., & Babcock, R. (2016). Coral reproduction in Western Australia. Peerj*,* 4, e2010. doi:10.7717/peerj.2010

Harrison, P. L., & Wallace, C. C. (1990). Reproduction, dispersal and recruitment of scleractinian corals. In Z. Dubinsky (Ed.), Ecosystems of the World: coral reefs (pp. 133-207). Amsterdam: Elsevier Publishers.

Okubo, N., Isomura, N., Motokawa, T., & Hidaka, M. (2007). Possible Self-Fertilization in the Brooding Coral Acropora (Isopora) brueggemanni. Zoological Science*,* 24, 277-280. doi:10.2108/zsj.24.277

Richards, Z., Delbeek, J. C., Lovell, E., Bass, D., Aeby, G., & Reboton, C. (2008). *Isopora brueggemanni*.The IUCN Red List of Threatened Species 2008: e.T133182A3618783. doi:10.2305/IUCN.UK.2008.RLTS.T133182A3618783.en

Richards, Z., Sampey, A., & Marsh, L. (2014). Kimberley marine biota. Historical data: scleractinian corals. Records of the Western Austra lian Museum Supplement*,* 84, 111-132.

Richards, Z., Garcia, R. A., Wallace, C. C., Rosser, N. L., & Muir, P. R. (2015). A diverse assemblage of reef corals thriving in a dynamic intertidal reef setting (Bonaparte Archipelago, Kimberley, Australia). Plos One*,* 10, e0117791.

Thomas, L., Underwood, J. N., Adam, A. A. S., Richards, Z. T., Dugal, L., Miller, K. J., & Gilmour, J. P. (2020). Contrasting patterns of genetic connectivity in brooding and spawning corals across a remote atoll system in northwest Australia. Coral Reefs*,* 39, 55-60. doi:10.1007/s00338-019-01884-8

Underwood, J. N., Smith, L. D., van Oppen, M. J. H., & Gilmour, J. P. (2007). Multiple scales of genetic connectivity in a brooding coral on isolated reefs following catastrophic bleaching. Molecular Ecology*,* 16, 771-784.

Underwood, J. N., Smith, L. D., van Oppen, M. J. H., & Gilmour, J. P. (2009). Ecologically relevant dispersal of a brooding and a broadcast spawning coral at isolated reefs: implications for managing community resilience. Ecological Applications*,* 19, 18-29.

Underwood, J. N., Miller, K. J., Puotinen, M., & Gilmour, J. P. (2018). Genetic signatures through space, time and multiple disturbances in a ubiquitous brooding coral. Molecular Ecology*,* in press.

van Oppen, M. J. H., Peplow, L. M., Kininmonth, S., & Berkelmans, R. (2011). Historical and contemporary factors shape the population genetic structure of the broadcast spawning coral, *Acropora millepora*, on the Great Barrier Reef. Molecular Ecology*,* 20, 4899-4914. doi:10.1111/j.1365-294X.2011.05328.x

Veron, J. J., & Marsh, L. J. (1988). Hermatypic corals of Western Australia. Records and annotated species list. . Records of the Western Australian Museum. Supplement.*,* 29, 1-136.
