# Supplementary material for: Extreme seascape drives local recruitment and genetic divergence in brooding and spawning corals in remote north‐west Australia
Source: Evol Appl. 2020 Jun 22;13(9):2404–21. doi: 10.1111/eva.13033 (PMC7513722; doi:10.1111/eva.13033)
Supplement: Supplementary file 2 — Appendix B [file EVA-13-2404-s002.docx]

# Appendix B: Diversity Array Technologies (DArT) SNP development and QC in *Acropora aspera* and *Isopora brueggemanni*

Genome-wide single nucleotide polymorphism (SNP) data were generated at Diversity Arrays Technology (DArT) with DArTseq methodology using the next generation sequencing platform. DArTseq represents a new implementation of sequencing of complexity reduced representations (Altshuler et al., 2000) and recent applications of this concept using the next generation sequencing platforms (Elshire et al., 2011). Detailed protocols are provided in Kilian et al. (2012), and examples of recent applications are Cruz et al. (2013), Raman et al. (2014) and Pazmino et al. (2017). The method is conceptually similar to RAD-seq methods (Baird et al., 2008), but in comparison, the generation of restriction fragments with appropriate adapters is more straightforward during the complexity reduction stage, therefore there is a high degree of qualitative and quantitative reproducibility in sampling genomic fragments. A subsample of six to eight individuals from 12 sites (n = 94) spread across the entire sample area (to avoid ascertainment bias) was used to optimise the DArTseq methodology. Four methods of complexity reduction were tested in corals (data not presented) to select the most appropriate method based on both the size of the representation and the fraction of a genome selected for assays, and the PstI-HpaII method was selected. DNA samples were processed in digestion/ligation reactions principally as per Kilian et al (2012) but replacing a single PstI-compatible adaptor with two different adaptors corresponding to two different Restriction Enzyme (RE) overhangs. The PstI-compatible adapter was designed to include Illumina flowcell attachment sequence, sequencing primer sequence and “staggered”, varying length barcode region, similar to that reported by Elshire et al. (2011). Reverse adapter contained flowcell attachment region and HpaII-compatible overhang sequence.

Only “mixed fragments” (PstI-HpaII) are effectively amplified in 30 rounds of PCR using the following reaction conditions: PCR conditions consisted of an initial denaturation at 94 °C for 1 min followed by 30 cycles of 94 °C for 20 sec, 58 °C for 30 sec and 72 °C for 45 sec, with a final extension step at 72 °C for 7 min. After PCR, equimolar amounts of amplification products from each sample of the 96-well microtiter plate were bulked and applied to c-Bot (Illumina) bridge PCR followed by sequencing on Illumina Hiseq2500. The sequencing (single read) was run for 77 cycles.

Once optimised, the above method was used to generate sequences from the entire collection, and then processed using proprietary DArT analytical pipelines. In the primary pipeline the FASTQ files are first processed to filter away poor quality sequences, applying more stringent selection criteria to the barcode region compared to the rest of the sequence. In that way the assignments of the sequences to specific samples carried in the “barcode split” step are very reliable. Approximately 2,500,000 (+/- 7%) sequences per barcode/sample were used in marker calling. Finally, identical sequences were collapsed into “fastqcall files”, which were groomed using DArT’s proprietary algorithm that corrects low quality bases from singleton tags using collapsed tags with multiple members as a template. These files are used in the secondary pipeline for DArT PL’s proprietary SNP calling algorithms (DArTsoft-14). All tags from all libraries were clustered using DArT PL’s C++ algorithm, followed by parsing of the clusters into separate SNP loci using a range of technical parameters, especially the balance of read counts for the allelic pairs. Additional selection criteria were added to the algorithm based on analysis of thousands of control crosses in large diversity of organisms. This allowed for testing for Mendelian distribution of alleles in these populations and facilitated selection of technical parameters discriminating true allelic variants from paralogous and contaminating viral and/or bacterial sequences.

195 *Isopora* samples and 126 *Acropora* samples were genotyped twice as technical replicates and scoring consistency was used as the main selection criteria for high quality/low error rate markers, and loci with reproducibility less than 0.94 were excluded. The call quality of the initial SNP data set was further assured by setting a cut-off of read depth per locus (coverage) < 7, call rate >0.35, minimum allele frequency > 0.00075 for *Isopora* and *> 0*.0017 for *Acropora*. Sequences were blasted on GenBank to check for general contamination and endosymbionts including genomes and transcriptomes of the symbiotic zooxanthellae, *Symbiodinium*, which lives in coral host tissue. No sequences aligned to the *Symbiodinium* genome for *Isopora*, while four sequences from *Acropora* aligned to the *Symbiodinium* genome with E-values between 1.36E^-19^ and 2.73E^-27^ and these were removed from downstream analysis. The primary data set comprised 23,165 SNPS for *I. brueggemanni* and 34,304 SNPS for *A. aspera.*

Altshuler, D., Pollara, V. J., Cowles, C. R., Van Etten, W. J., Baldwin, J., Linton, L., & Lander, E. S. (2000). An SNP map of the human genome generated by reduced representation shotgun sequencing. Nature*,* 407, 513-516.

Baird, N. A., Etter, P. D., Atwood, T. S., Currey, M. C., Shiver, A. L., Lewis, Z. A., Selker, E. U., Cresko, W. A., & Johnson, E. A. (2008). Rapid SNP Discovery and Genetic Mapping Using Sequenced RAD Markers. Plos One*,* 3. doi:10.1371/journal.pone.0003376

Cruz, V. M. V., Kilian, A., & Dierig, D. A. (2013). Development of DArT Marker Platforms and Genetic Diversity Assessment of the US Collection of the New Oilseed Crop Lesquerella and Related Species. Plos One*,* 8. doi:10.1371/journal.pone.0064062

Elshire, R. J., Glaubitz, J. C., Sun, Q., Poland, J. A., Kawamoto, K., Buckler, E. S., & Mitchell, S. E. (2011). A Robust, Simple Genotyping-by-Sequencing (GBS) Approach for High Diversity Species. Plos One*,* 6. doi:10.1371/journal.pone.0019379

Kilian, A., Wenzl, P., Huttner, E., Carling, J., Xia, L., Blois, H., Caig, V., Heller-Uszynska, K., Jaccoud, D., Hopper, C., Aschenbrenner-Kilian, M., Evers, M., Peng, K., Cayla, C., Hok, P., & Uszynski, G. (2012). Diversity Arrays Technology: A Generic Genome Profiling Technology on Open Platforms. In F. Pompanon & A. Bonin (Eds.), Data Production and Analysis in Population Genomics (Vol. 888, pp. 67-89): Humana Press.

Pazmino, D. A., Maes, G. E., Simpfendorfer, C. A., Salinas-de-Leon, P., & van Herwerden, L. (2017). Genome-wide SNPs reveal low effective population size within confined management units of the highly vagile Galapagos shark (Carcharhinus galapagensis). Conservation Genetics*,* 18, 1151-1163. doi:10.1007/s10592-017-0967-1

Raman, H., Raman, R., Kilian, A., Detering, F., Carling, J., Coombes, N., Diffey, S., Kadkol, G., Edwards, D., McCully, M., Ruperao, P., Parkin, I. A. P., Batley, J., Luckett, D. J., & Wratten, N. (2014). Genome-Wide Delineation of Natural Variation for Pod Shatter Resistance in Brassica napus. Plos One*,* 9. doi:10.1371/journal.pone.0101673
