# Supplementary material for: Extreme seascape drives local recruitment and genetic divergence in brooding and spawning corals in remote north‐west Australia
Source: Evol Appl. 2020 Jun 22;13(9):2404–21. doi: 10.1111/eva.13033 (PMC7513722; doi:10.1111/eva.13033)
Supplement: Supplementary file 3 — Appendix C [file EVA-13-2404-s003.docx]

# Appendix C: Clonality Analysis

In many branching corals, sexual reproduction is often supplemented by asexual vegetative fragmentation, whereby branches break off through physical disturbance but re-attach to the substrate and continue to grow (Knowlton et al., 1981; Lirman, 2000; Smith & Hughes, 1999; Wallace, 1985).

To establish whether colonies were clones produced by vegetative fragmentation, we used the technical replicates to determine a threshold of maximum genetic distance (based on hamming distance Appendix B) between the two genotypes of each repeat pair, and identified clones as samples with genotypic distance below this threshold. For colonies that were identified as clones (ramets), all but one individual was removed from the data, yielding a final data set comprising unique colonies (genets). All technical replicates exhibited a hamming distance of < 0.01 for *A. aspera* and < 0.02 for *I. brueggemanni,* resulting in the removal of 207 and 45 samples respectively from these data sets. All clones were collected at the same site.

The extent of clonal proliferation in A*cropora* asp-c varied among the sites. Mean genotypic richness across all sites was relatively low (0.61), but ranged from 0.25 to 1.00 (Table 1). Clones were most abundant at White Island, Bathurst N Satellite, Bathurst E Satellite, Bowles Rock and Pope Island where 60% - 70% of all samples were clones (Table 1). At Bowles Rock and Pope Island, many clones were represented by a several genets (separated by a few metres), while the collection at Bathurst N Satellite was dominated by one common clone (separated by tens of metres). Thus, clonal propagation appears to be important for *Acropora* asp-c at some sites in the Kimberley. This result is consistent with other work in northwest Australia, which indicates the establishment of vegetative fragments in *Acropora tenuis* that aid recovery after tropical storms on the inshore reefs with shallow depth gradients is common (Underwood, 2009). The relatively high levels of clonality in *Acropora* asp-c were not however, associated with reduced gene diversity at these sites (Figure 3). Therefore, even for those reefs where vegetative fragments are abundant, sexual reproduction continues to be important for maintenance of genetic variation in these populations.

Genotypic richness was generally high in *I. brueggemanni*, with an average of 0.93 across all sites (Table 2). Nine sites were comprised entirely of unique genets, although one site (Hedley Island) had a much lower genotypic richness than all other sites (0.39; Table 2). Therefore, vegetative fragmentation appears to be much less common in the more robust branching growth form of I*. brueggemanni*, with high genotypic richness at all the sites except one, suggesting that sexually produced larvae dominate reproduction in these populations.

*References*

Knowlton, N., Lang, J. C., Rooney, M. C., & Clifford, P. (1981). Evidence for delayed mortality in hurricane-damaged Jamaican staghorn corals. *Nature, 294*(5838), 251-252.

Lirman, D. (2000). Fragmentation in the branching coral *Acropora palmata* (Lamarck): growth, survivorship, and reproduction of colonies and fragments. *Journal of Experimental Marine Biology and Ecology, 251*(1), 41-57.

Smith, L. D., & Hughes, T. P. (1999). An experimental assessment of survival, re-attachment and fecundity of coral fragments. *Journal of Experimental Marine Biology and Ecology, 235*(1), 147-164.

Underwood, J. N. (2009). Genetic diversity and divergence among coastal and offshore reefs in a hard coral depend on geographic discontinuity and oceanic currents. *Evolutionary Applications, 2* 222-233.

Wallace, C. C. (1985). Reproduction, recruitment and fragmentation in nine sympatric species of the coral genus *Acropora*. *Marine Biology, 88*(3), 217-233.
