# Supplementary material for: Extreme seascape drives local recruitment and genetic divergence in brooding and spawning corals in remote north‐west Australia
Source: Evol Appl. 2020 Jun 22;13(9):2404–21. doi: 10.1111/eva.13033 (PMC7513722; doi:10.1111/eva.13033)
Supplement: Supplementary file 4 — Appendix D [file EVA-13-2404-s004.docx]

**Appendix D Additional results of genomic analysis for *Acropora aspera* and *Acropora* asp-c lineage**


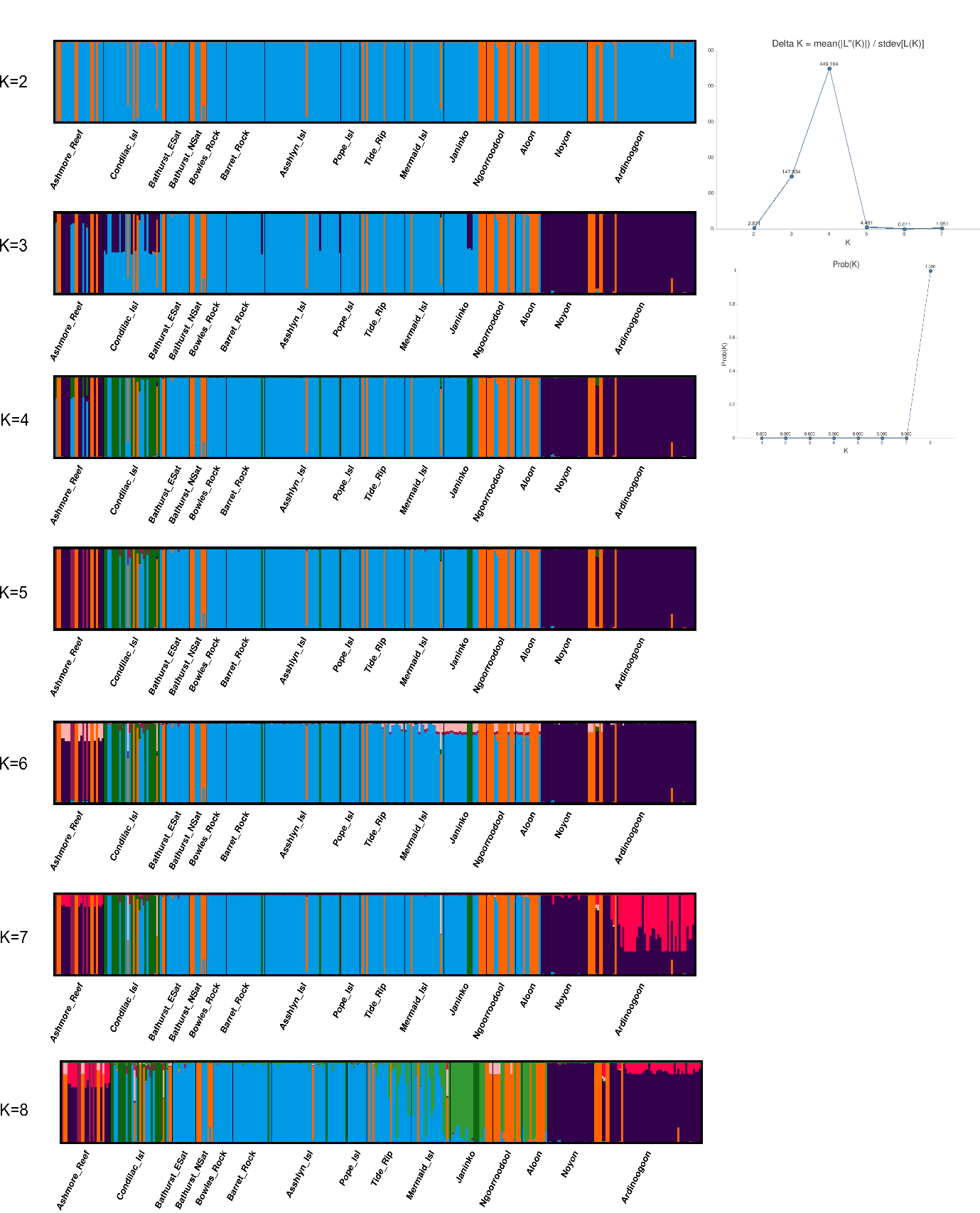


Figure D1 Barplots from STRUCTURE analysis showing membership coefficients from of the entire *Acropora aspera* collection using NORPRIOR and correlated allele frequency model for K = 2 to 8 using the stringent data set of 585 SNP loci. Major modes calculated in CLUMPAK are presented. Insets shows plots of ΔK and Ln (Pr(X|K) for increasing K.


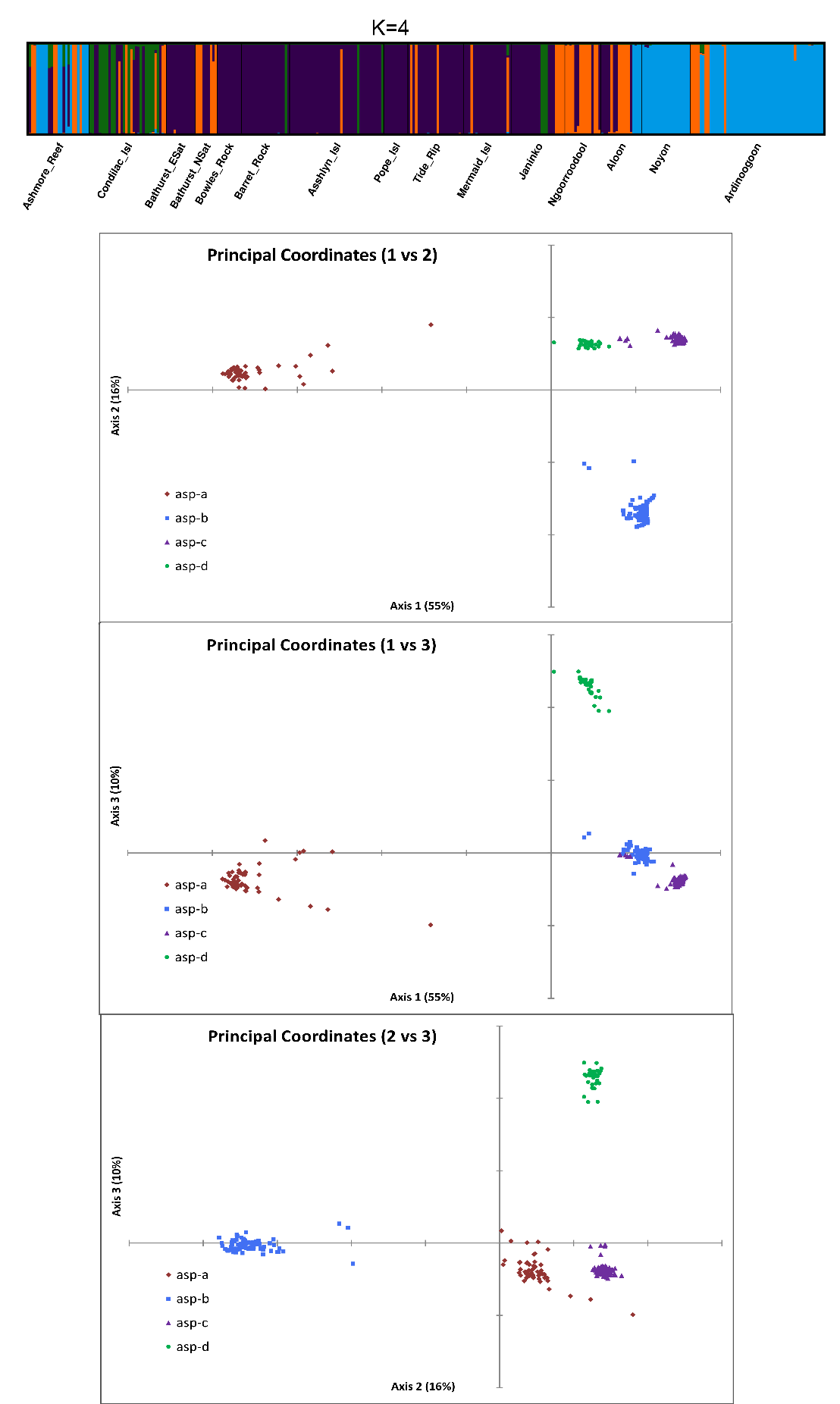


Figure D2 Clustering analysis results from the entire Acropora aspera collection using the relaxed data set of 3,698 SNP loci produced by the relaxing filters. Upper panel shows barplot of membership coefficients of individual corals calculated with STRUCTURE v2.3 with no prior information for K = 4. CLUMPAK calculated this plot from 10/10 runs and a similarity score = 0.999 and mean (LnProb) = -270084. Lower panels show Principal Coordinates Analysis (PCoA) of all three axes calculated from individual pairwise genotypic distance. Individuals are colour coded according to the clusters assigned by the STRUCTURE analysis. Percentage of variation explained by each axis is given in brackets.


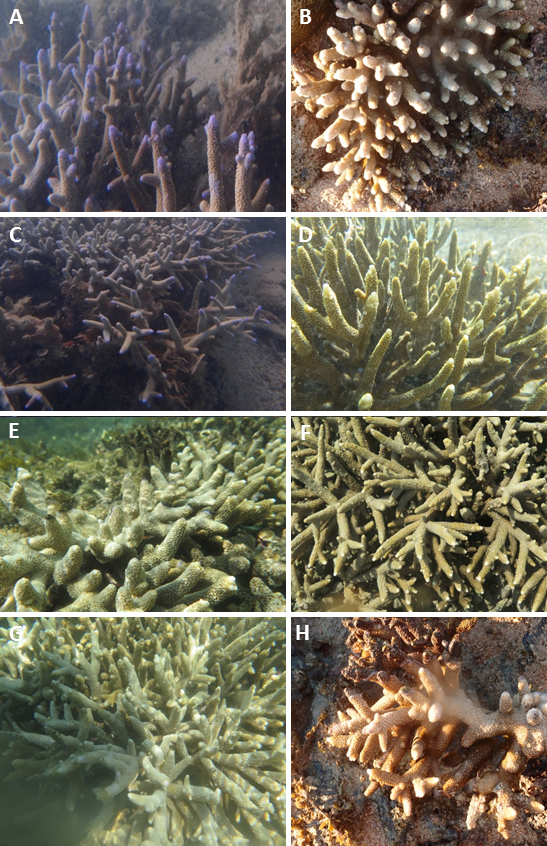


Figure D3 Photos of colonies belonging to each of the four *Acropora aspera* genetic lineages showing that macro-morphological variation between lineages appears to be as great as the variation within lineages. A. *Acropora* asp-a collected from Ardinoogoon (Shenton Bluff); B. *Acropora* asp-a collected from Aloon (Jackson Island); C. *Acropora* asp-b collected from Noyon; D. *Acropora* asp-b collected form Ardinoogoon (Shenton Bluff); E. *Acropora* asp-c collected from Janinko (Sunday Island); F. *Acropora* asp-c collected from Mermaid Island; G. *Acropora* asp-d collected from Janinko (Sunday Island); H. *Acropora* asp-d collected form Aloon (Jackson Island).

Table D1 Pairwise *F*_ST_ values among lineages of corals from the entire *Acropora aspera* collection identified with 3,698 SNP from the relaxed filtering.

|  | **asp-a** | **asp-b** | **asp-c** | **asp-d** |
| --- | --- | --- | --- | --- |
| **asp-a** | 0.000 |  |  |  |
| **asp-b** | 0.637 | 0.000 |  |  |
| **asp-c** | 0.721 | 0.511 | 0.000 |  |
| **asp-d** | 0.629 | 0.524 | 0.578 | 0.000 |

Figure D4 Estimates of gene diversity of the four *Acropora aspera* clusters based on expected heterozygosity (± standard errors) and 585 loci.


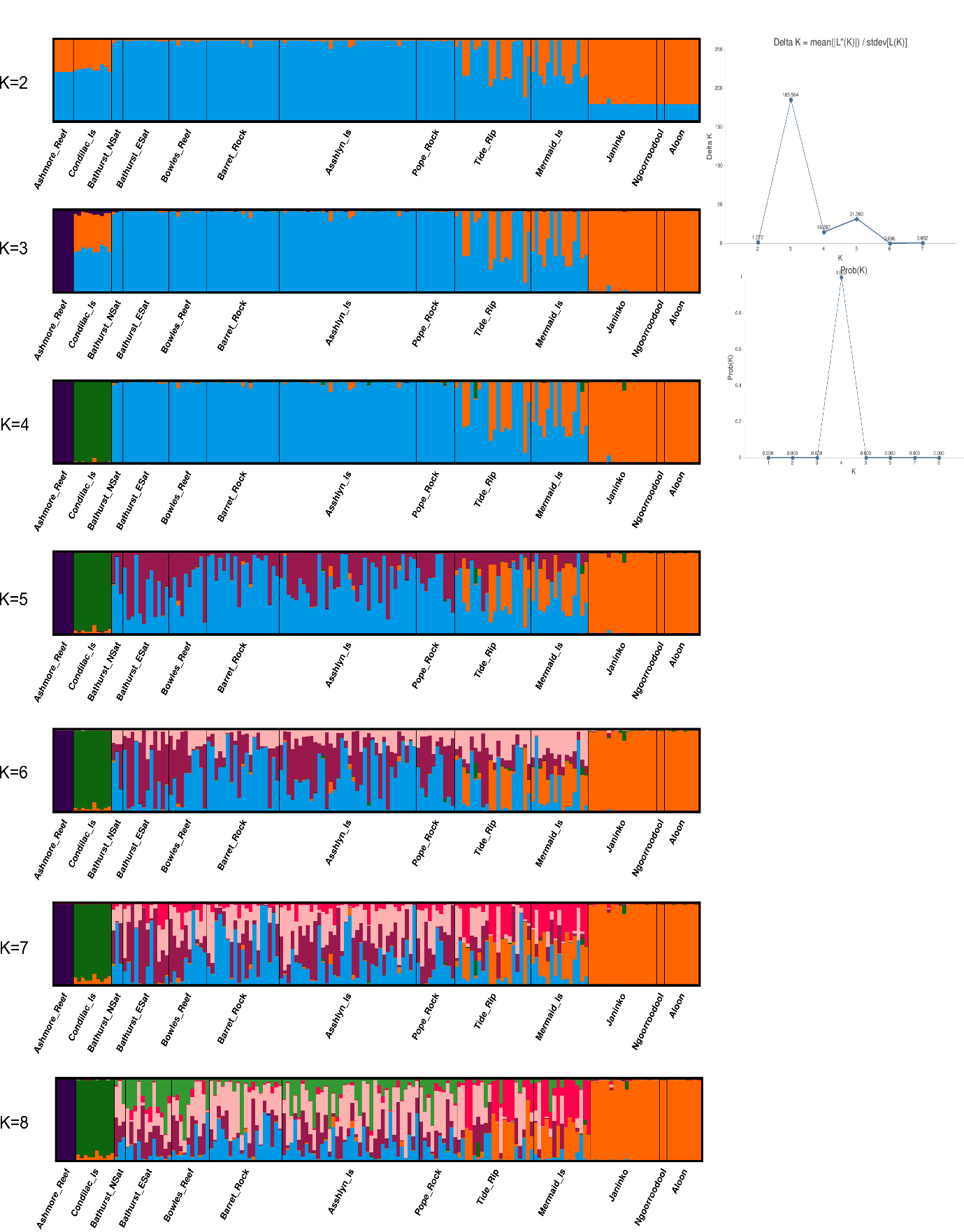


Figure D5 Barplots from NOPRIOR runs in STRUCTURE showing membership coefficients for K = 2 to 8 of colonies in the *Acropora* asp-c lineage. Major modes calculated in CLUMPAK are presented. Plot on right shows ΔK for increasing K from STRUCTURE analyses of the Acropora asp-c lineage with prior information on sampling location.


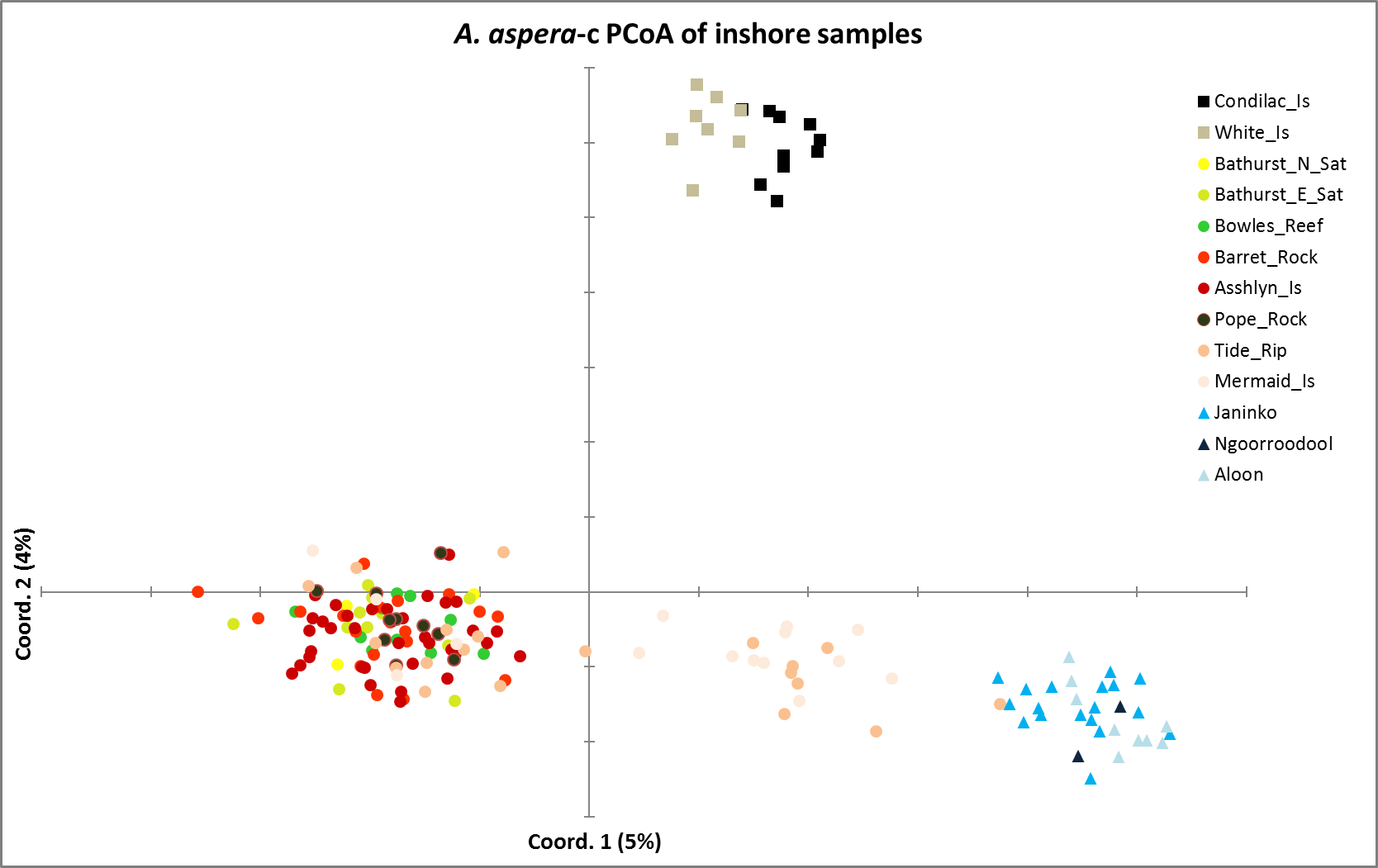


Figure D6 Principal Coordinates analysis (PCoA) calculated form individual pairwise genotypic distance of corals from the Acropora asp-c lineage from the inshore Kimberley only (i.e. Ashmore Reef samples are excluded). Percentage of variation explained by each axis is given in brackets.

Table D2Pairwise F_ST_ estimates between sites for *Acropora* asp-c in the Kimberley below diagonal, and P-values based on 999 permutations are shown above diagonal. Sites with sample size <4 were excluded.

Table D3 Results of AMOVA that partitioned genetic variation among systems, among sites within systems and among all sites within the *Aspera* asp-c lineage. Analysis involved all sites, and only those sites with n ≥ 9. All estimates of differentiation were significant at P < 0.05.
