# Supplementary material for: Extreme seascape drives local recruitment and genetic divergence in brooding and spawning corals in remote north‐west Australia
Source: Evol Appl. 2020 Jun 22;13(9):2404–21. doi: 10.1111/eva.13033 (PMC7513722; doi:10.1111/eva.13033)
Supplement: Supplementary file 5 — Appendix E [file EVA-13-2404-s005.docx]

Appendix E Additional results of genomic analysis of *Isopora brueggemanni*


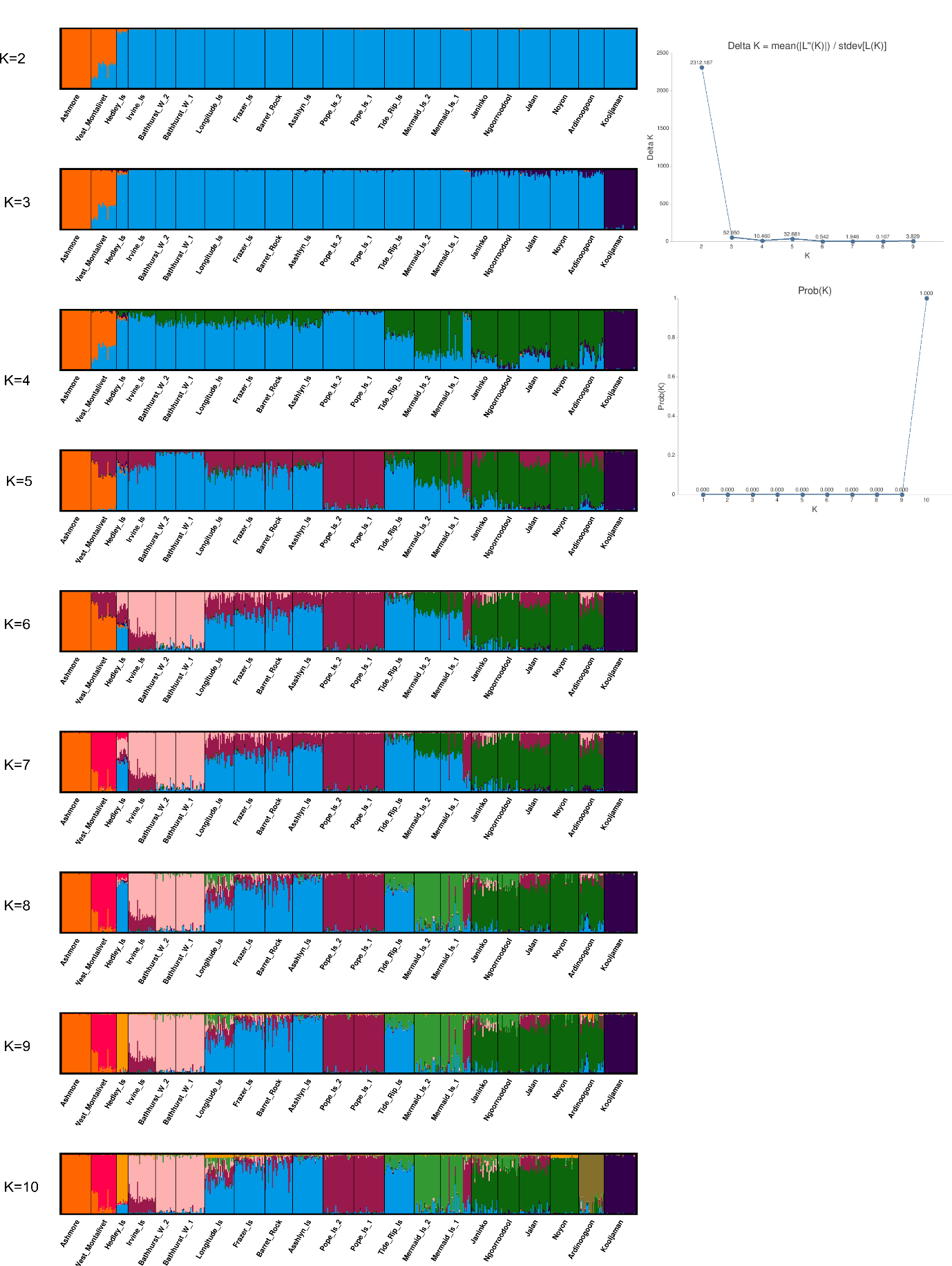


Figure E1 Barplots from STRUCTURE analysis using the NOCPRIOR model showing membership coefficients for K = 2 to 10 of the entire *Isopora brueggemanni* collection. Major modes calculated in CLUMPAK are presented. Insets shows plots of ΔK and Ln (Pr(X|K) for increasing K plot of ΔK for increasing K shown on right.


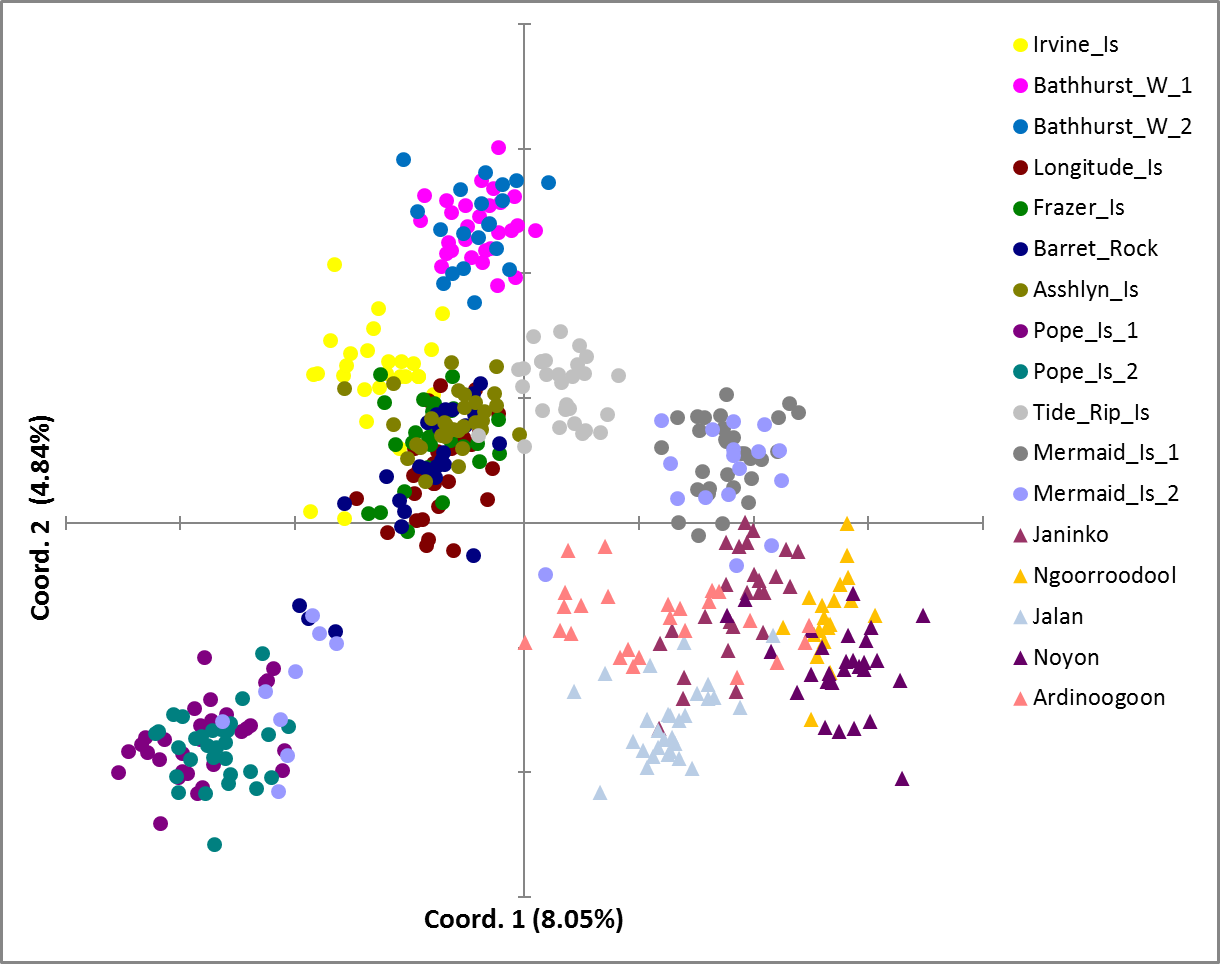


Figure E2 Principal Coordinates analysis (PCoA) calculated form individual pairwise genotypic distance of *I. brueggemanni* samples collected from the Dampier Peninsula and the Buccaneer Archipelago. Individuals are colour coded according to location, and the Buccaneer Archipelago samples are represented by circles and Dampier Peninsula samples by triangles. Note that Kooljaman was excluded to elucidate local scale patterns of genetic structure. Percentage of variation explained by each axis is given in brackets.

Table E1 Pairwise F_ST_ estimates between sites for *I. brueggemanni* in the Kimberley below diagonal, and P-values significance based on 999 permutations are shown above diagonal.
